# Effect of metabolic status on response to SIV infection and antiretroviral therapy in nonhuman primates

**DOI:** 10.1172/jci.insight.181968

**Published:** 2024-08-08

**Authors:** Gabriela M. Webb, Kristin A. Sauter, Diana Takahashi, Melissa Kirigiti, Lindsay Bader, Sarah R. Lindsley, Hannah Blomenkamp, Cicely Zaro, Molly Shallman, Casey McGuire, Heather Hofmeister, Uriel Avila, Cleiton Pessoa, Joseph M. Hwang, Allyson McCullen, Matthew Humkey, Jason Reed, Lina Gao, Lee Winchester, Courtney V. Fletcher, Oleg Varlamov, Todd T. Brown, Jonah B. Sacha, Paul Kievit, Charles T. Roberts

**Affiliations:** 1 Division of Pathobiology and Immunology, and; 2 Division of Metabolic Health and Disease, Oregon National Primate Research Center (ONPRC), Beaverton, Oregon, USA.; 3 Knight Cancer Institute, Oregon Health and Science University, Portland, Oregon, USA.; 4 Antiviral Pharmacology Laboratory, Center for Drug Discovery, University of Nebraska Medical Center, Omaha, Nebraska, USA.; 5 Division of Endocrinology, Diabetes and Metabolism, Johns Hopkins University, Baltimore, Maryland, USA.; 6 Division of Reproductive and Developmental Sciences, Oregon National Primate Research Center (ONPRC), Beaverton, Oregon, USA.

**Keywords:** AIDS/HIV, Metabolism, Adipose tissue, Glucose metabolism, Obesity

## Abstract

Current antiretroviral therapy (ART) regimens efficiently limit HIV replication, thereby improving the life expectancy of people living with HIV; however, they also cause metabolic side effects. The ongoing obesity epidemic has resulted in more people with metabolic comorbidities at the time of HIV infection, yet the effect of preexisting metabolic dysregulation on infection sequelae and response to ART is unclear. Here, to investigate the impact of preexisting obesity and insulin resistance on acute infection and subsequent long-term ART, we infected a cohort of lean and obese adult male macaques with SIV and administered ART. The responses of lean and obese macaques to SIV and ART were similar with respect to plasma and cell-associated viral loads, ART drug levels in plasma and tissues, SIV-specific immune responses, adipose tissue and islet morphology, and colon inflammation, with baseline differences between lean and obese groups largely maintained. Both groups exhibited a striking depletion of CD4^+^ T cells from adipose tissue that did not recover with ART. However, differential responses to SIV and ART were observed for body weight, omental adipocyte size, and the adiponectin/leptin ratio, a marker of cardiometabolic risk. Thus, obesity and insulin resistance had limited effects on multiple responses to acute SIV infection and ART, while several factors that underlie long-term metabolic comorbidities were influenced by prior obesity and insulin resistance. These studies provide the foundation for future investigations into the efficacy of adjunct therapies such as metformin and glucagon-like peptide-1 receptor agonists in the prevention of metabolic comorbidities in people living with HIV.

## Abstract 2

Currrent HIV therapy can result on adverse effects such as obesity and prediabetes. This study used a nonhuman primate model of HIV infection and antiviral therapy to determine if pre-existing obesity altered the effects of infection and therapy.

## Introduction

Modern antiretroviral therapy (ART) has transformed HIV/AIDS from a usually fatal disease to a manageable chronic condition. People initiating ART early after infection can achieve a near-normal life expectancy, although still 5–10 years shorter than persons without HIV (1, 2). As a consequence, however, age-associated chronic diseases such as obesity, diabetes, and cardiovascular disease have increased in people living with HIV (PLWH). While early ART regimens resulted in lipodystrophy-induced insulin resistance, modern ART regimens, particularly those containing integrase strand transfer inhibitors, are reported to increase the risk of overall adiposity and, in turn, insulin resistance, prediabetes, and frank type 2 diabetes (3–15), thus adding to the age-related burden of these disorders. These metabolic comorbidities and their associated costs have exhibited significant increases in recent years (16, 17) and represent an ongoing public health issue (18). ART-associated metabolic dysfunction is thought to reflect persistent HIV-induced chronic inflammation that is incompletely resolved by ART (19–23), despite efficient reduction of viremia. The significance of ART-associated obesity and metabolic dysfunction is heightened by the growing appreciation that white adipose tissue (WAT) plays an important role in the metabolic comorbidities associated with ART by virtue of its control of lipid metabolism as well as in systemic glucose homeostasis through the secretion of important adipocytokines (24, 25). In addition, WAT has recently emerged as an important latent viral reservoir (26–30) and a potential site of sequestration of lipophilic ART components (27). These findings place WAT at a critical juncture of ART-associated metabolic comorbidities and the ART-suppressed latent reservoir. WAT morphology, cellular (adipocyte and immune cell) profiles, and function are specifically affected by HIV and ART (31–37).

Although significant attention has been focused on postinfection/ART-associated comorbidities, there is a paucity of data on the contribution of preexisting conditions, in particular obesity-related metabolic disease, to disease course, ART efficacy, and the prevalence and severity of ART-associated comorbidities. This is an increasingly important issue in light of the global obesity epidemic and worldwide increases in the rates of type-2 diabetes and diabetic complications. Specifically, obesity and prediabetes (and associated insulin resistance) continue to increase and are major contributors to overall morbidity and mortality (38–42). This, in turn, is driving the continued increase in type-2 diabetes (43, 44). Thus, the dramatic increase in obesity and metabolic disease in both the general and at-risk populations in the US as well as worldwide has resulted in an increased number of individuals who are obese at the time of infection. In fact, several studies have reported increased incidence of obesity in both males and females, including adolescents, with newly diagnosed HIV (45–48). It is reasonable to assume that these individuals were obese at the time of infection rather than rapidly becoming obese prior to diagnosis.

Macaque species have long been primary preclinical models for HIV infection and disease due to their susceptibility to the primate lentivirus SIV, with immunopathology and disease sequelae that mirror HIV infection in humans (49–51). SIV models have been developed primarily in the rhesus macaque using various natural SIV strains and unique recombinant viruses, such as SHIV, for intervention studies. Macaques also exhibit many other aspects of physiology that closely resemble human biology and that are particularly relevant to HIV comorbidities. Specifically, macaques exhibit multiple anatomically distinct white and brown adipose tissue depots (52, 53), primate-specific aspects of pancreatic islet structure and function (54), and susceptibility to Western-style diet-induced obesity, diabetes, and cardiovascular disease (55–57). In this study, we exploited the rhesus macaque model of SIV infection and ART to assess the effect of preexisting obesity/insulin resistance on viral dynamics and efficacy of ART suppression as well as an array of immunological and metabolic parameters. We were specifically interested in the effects of SIV infection and subsequent ART on the appearance of new metabolic comorbidities in lean animals and the potential exacerbation of existing metabolic dysfunction in obese animals. The overall rationale for this experimental approach was that the identification of specific parameters affected by SIV and ART, and that are differentially affected in obese animals, could reveal potential opportunities for targeted preventative or therapeutic approaches to reduce the burden of metabolic comorbidities in PLWH. Possible interventions could include agents such as metformin or GLP-1 receptor agonists.

## Results

### SIV infection and ART differentially affect BW but not viral dynamics in lean and obese animals.

**Figure 1:**
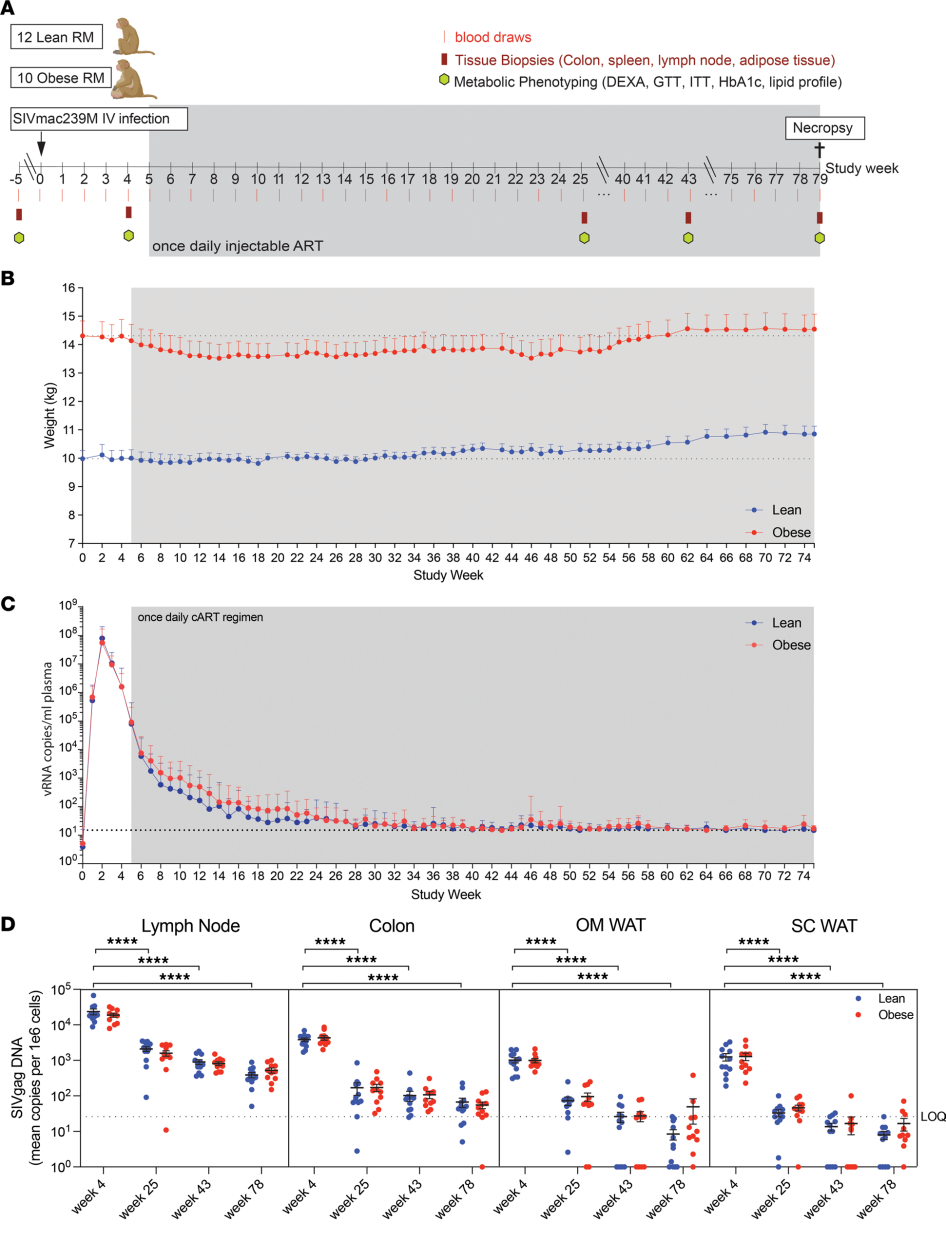
Experimental timeline, BW changes, and plasma RNA and cell-associated viral DNA dynamics during SIV infection and ART suppression. (**A**) Schedule of experimental procedures and assessments. (**B**) BW in lean (*n* = 12) and obese (*n* = 10) groups over the study time course. Horizontal dotted lines indicate average baseline BW. Insert shows difference in baseline average BW. (**C**) SIV plasma RNA copies per mL over the study time course. Limit of quantification (LOQ) is denoted by the horizontal dotted line. (**D**) Cell-associated SIV DNA in peripheral lymph node, colon, and the SVF of OM and SC WAT over the study time course. In all figures, data points for lean animals are in blue and those for obese animals are in red. Significance was determined by ordinary 1-way ANOVA with Tukey’s multiple comparison test. ****P < 0.0001. All data are means ± SEM.

To explore the effects of obesity and insulin resistance on the response to SIV infection and long-term ART, we infected 12 lean and 10 obese rhesus macaques with SIVmac239M, allowed infection to proceed for 5 weeks, and then initiated daily ART for 74 weeks. Figure 1A depicts the longitudinal experimental design and the schedule of sampling and assessments, while Table 1 outlines the baseline characteristics of the lean and obese groups. The obese group exhibited significantly greater BW, body fat, fasting insulin and C-peptide, Homeostasis Model Assessment for Insulin Resistance (HOMA-IR), hemoglobin A1c (HbA1c), and iv glucose tolerance test–based (ivGTT-based) measures of glucose homeostasis (glucose, insulin, and C-peptide AUC), thus establishing the distinct baseline metabolic status of the 2 experimental groups.

As shown in Figure 1B, BW changes in response to infection and ART differed between the lean and obese groups, with lean animals maintaining their baseline weight until complete suppression of plasma viral load at weeks 30–34 (Figure 1C), while the obese group lost weight at the initiation of ART and did not rebound until 58 weeks postinfection. Specifically, the average BW of animals in the lean group remained constant through week 30 and increased thereafter, resulting in an overall approximately 10% weight gain after 16 months of ART. In contrast, animals in the obese group exhibited a 5% decrease in BW over the first 15 weeks that was maintained through week 54, at which time this group exhibited an increase to slightly above baseline levels by 62 weeks. In spite of these differences, both groups exhibited similar rapid increases in plasma viral load after infection, peaking at 2 weeks and declining thereafter. After ART initiation at 5 weeks postinfection, plasma viral loads continued to decline until complete suppression was achieved at approximately 30–34 weeks postinfection in both groups (Figure 1C). A more sensitive plasma viral load assay was implemented after week 20 in the second cohort of animals, as described in the Methods section, but did not affect these conclusions. Of note, there was a trend toward a slower decay of plasma viremia in the obese group during weeks 6–24, but this was not statistically significant.

Cell-associated SIV DNA levels as a measure of the SIV reservoir were also determined in longitudinal biopsies of peripheral (inguinal or axillary) lymph node, colon, and omental (OM) and subcutaneous (SC) WAT. As shown in Figure 1D, pre-ART tissue-associated SIV DNA levels (week 4) were highest in lymph node and colon and exhibited significant declines following ART initiation, but levels remained detectable through week 78 after 16 months of ART (Supplemental Figure 1; supplemental material available online with this article; https://doi.org/10.1172/jci.insight.181968DS1). Initial SIV DNA levels in WAT depots were lower and also exhibited significant declines following ART. However, levels of cell-associated virus remained detectable through the end of the study at 78 weeks postinfection, with levels above the limit of quantification (LOQ) found almost exclusively in obese animals (Figure 1D and Supplemental Figure 1). As with plasma viral loads, no differences in the viral reservoir were seen between the lean and obese groups at any timepoint. Since cell-associated viral loads were assayed in the WAT stromovascular fraction (SVF), it was not possible to determine the specific WAT SVF cell type (i.e., immune cells versus endothelial cells versus preadipocytes) harboring persistent viral DNA or the presence of viral DNA in mature adipocytes.

### Tissue ART drug concentrations are similar in lean and obese animals.

**Figure 2:**
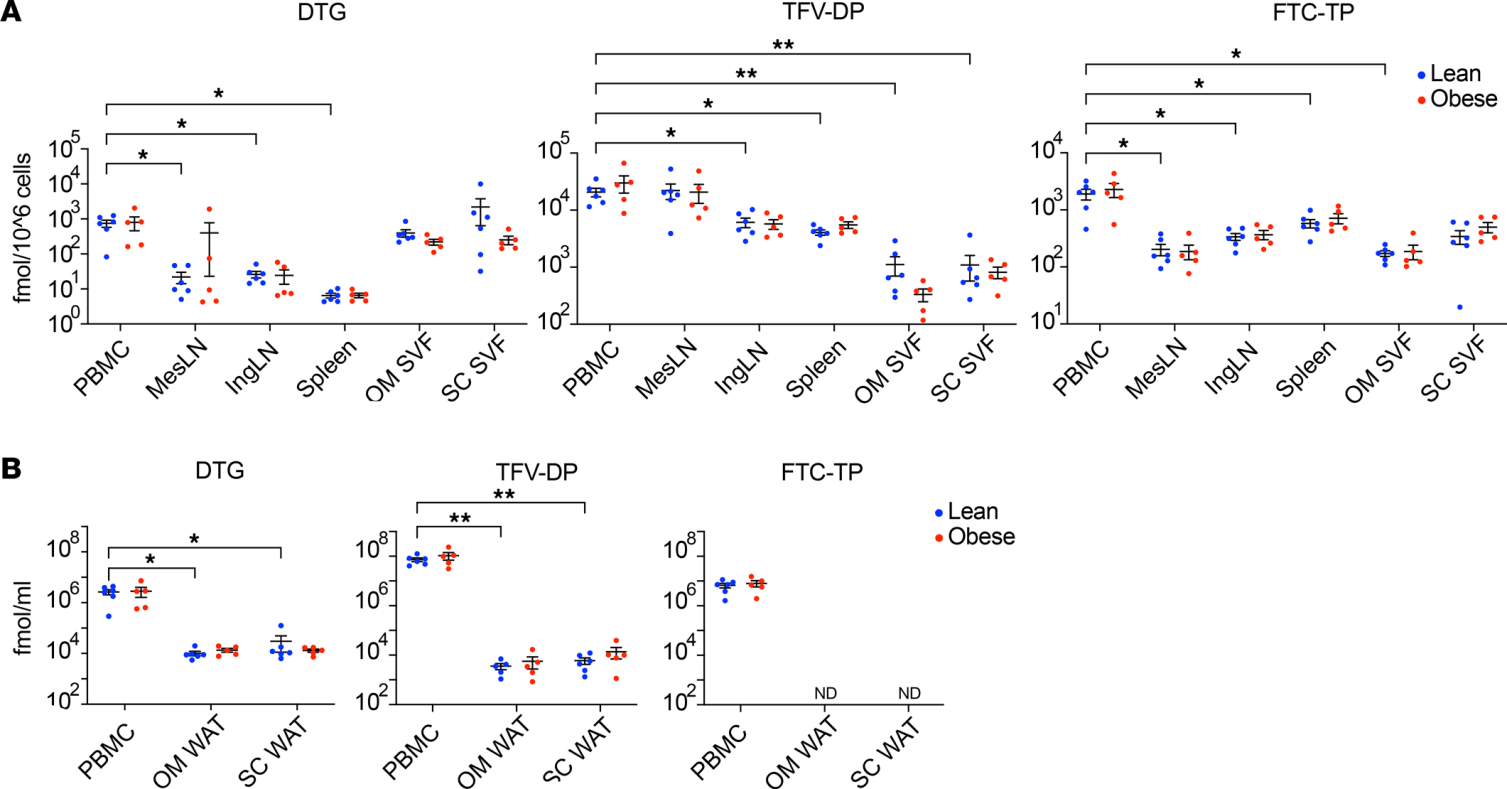
ART drug concentrations in tissues. (**A**) Average levels of dolutegravir (DTG), tenofovir diphosphate (TFV-DP), and emtricitabine triphosphate (FTC-TP) in peripheral blood mononuclear cells (PBMCs), dissociated cells from mesenteric lymph node (MesLN), inguinal lymph node (IngLN), spleen, and OM and SC WAT SVF cells (SC and OM SVF) at necropsy (week 72) (cohort 1 only). (**B**) DTG, TFV-DP, and FTC-TP levels in PBMCs and the lipid fraction of OM and SC WAT. Conversion of the levels of DTG, TFV-DP, and FTC-TP in fmol/1 × 10^6^ PBMCs to fmol/mL in panel **B** employed the average PBMC volume described by Simiele et al. (104). Data points for lean animals are in blue and those for obese animals are in red. Significance was determined by mixed-effects analysis with Tukey’s multiple comparison test. *P < 0.05; **P < 0.01. All data are means ± SEM.

Because previous reports suggested ART components might be sequestered in adipose tissue (58), we measured the intracellular concentrations of individual ART components in PBMCs and cells isolated from mesenteric and inguinal lymph nodes (LNs), spleen, and the SVF of SC and OM WAT at necropsy from 6 lean and 5 obese animals in the first cohort (1 obese macaque was lost due to procedure-associated mortality). As shown in Figure 2A, levels of the integrase strand transfer inhibitor dolutegravir (DTG) were higher in PBMCs than all other samples except SVF from SC and OM WAT, in which levels were similar to PBMCs. Concentrations of the nucleoside reverse transcriptase inhibitor tenofovir diphosphate (TFV-DP) were highest in PBMCs and mesenteric LNs, and concentrations of the nucleoside reverse transcriptase inhibitor emtricitabine triphosphate (FTC-TP) were highest in PBMCs versus LN, spleen, and WAT. There were no significant differences in ART drug concentrations in any tissue between the lean and obese groups. Since different ART components differ in their lipophilicity, we also assessed ART drug concentrations in PBMCs compared with the lipid fraction of SC and OM WAT adipocytes, both expressed as fmol/mL of cell volume to allow a more direct comparison. Since the unilocular lipid droplet represents more than 90% of the cell volume of a mature adipocyte, drug concentrations in the lipid fraction represent a reasonable estimate of the concentrations in the adipocyte component of WAT. As shown in Figure 2B, the concentrations of DTG and TFV-DP in PBMCs on this volume basis were significantly greater than the concentrations in the WAT lipid fraction, while FTC-TP concentrations were significant in PBMCs but undetectable in the WAT lipid fraction. As we determined the doses for all study animals based on BW, these data suggest that there is not significant sequestration of ART components in adipocytes. There were also no differences in the plasma concentrations of DTG, the TFV prodrug tenofovir disoproxil fumarate (TDF), and FTC in the lean and obese groups (data not shown).

### SIV and ART differentially affect T cell profiles in WAT versus whole blood and in different WAT depots.

Since WAT is increasingly recognized as an important latent viral reservoir by virtue of the presence of resident immune cells such as macrophages and CD4^+^ T cells, we examined WAT immune cell profiles by flow cytometry and compared them with parallel changes in peripheral immune cells during SIV infection and subsequent ART.

**Figure 3:**
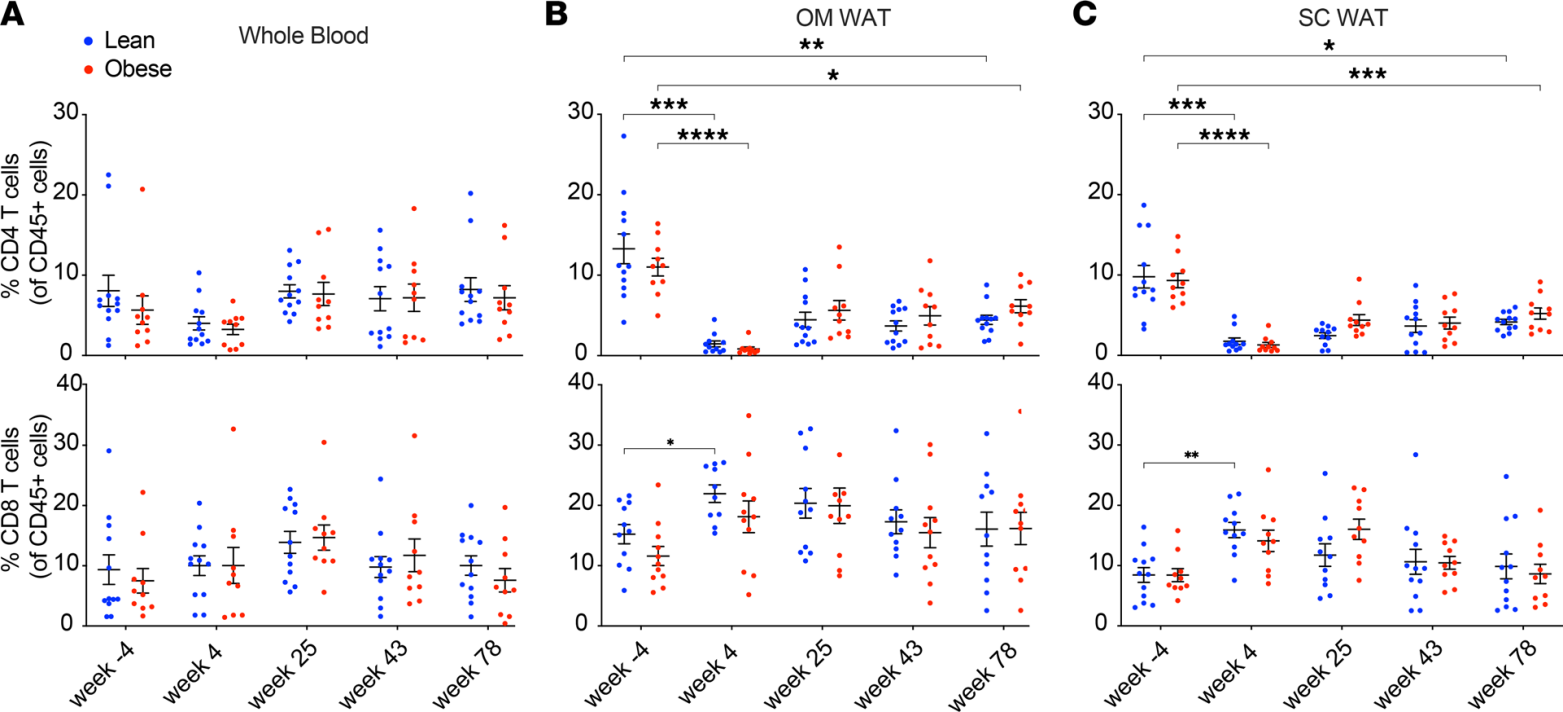
Effect of SIV infection and ART suppression on CD4^+^ and CD8^+^ T cell frequencies in whole blood and OM and SC WAT. Whole blood monocytes (**A**) and the stromovascular fraction of OM (**B**) and SC (**C**) WAT were analyzed by flow cytometry using the gating strategy and antibodies described in Supplemental Figure 12 and Supplemental Table 1. Data points for lean animals are in blue and those for obese animals are in red. Significance was determined by mixed-effects analysis with Tukey’s multiple comparison test. *P < 0.05; **P < 0.01; ***P < 0.001. All data are means ± SEM.

Changes in T cell profiles in whole blood and OM and SC WAT over the course of SIV infection and ART are shown in Figure 3. There were no significant changes in the frequencies of CD4^+^ or CD8^+^ T cells in whole blood. In contrast, there was a significant decrease in the frequencies of OM and SC WAT CD4^+^ cells following infection in both groups, with a recovery after ART that did not reach preinfection baseline levels. The frequencies of OM and SC WAT CD8^+^ T cells were increased after infection in both groups, which was significant only in the lean group. CD8^+^ T cell frequencies then decreased to baseline levels in both groups following long-term ART.

We also assessed the longitudinal changes in the frequencies of the T cell activation/proliferation markers CD69, CD38, and Ki67, as well as memory T cell subsets. Supplemental Figure 2 shows the longitudinal changes in CD38, CD69, and Ki67 CD4^+^ and CD8^+^ T cells. We evaluated CD69 expression as a tissue-resident T cell marker of early activation (Supplemental Figure 2A). As expected, CD69 expression was minimal on T cells in whole blood, whereas surface expression of CD69 on WAT CD4^+^ and CD8^+^ T cells was higher compared with whole blood. In OM WAT, CD69 CD4^+^ T cell frequency decreased in acute infection and rebounded to greater-than-baseline levels after ART. Expression of CD38, a hallmark of immune activation in HIV and SIV infection, was increased in CD4^+^ T cells in OM WAT and in CD8^+^ cells in SC WAT in both lean and obese groups after infection and returned to baseline levels with prolonged ART (Supplemental Figure 2B). Lastly, we monitored Ki67 expression, a marker of proliferation and activation (Supplemental Figure 2C). Both lean and obese groups exhibited significantly increased Ki67 expression at 4 weeks postinfection in both CD4^+^ and CD8^+^ T cells in SC WAT, indicating increased activation during acute infection. In contrast, in OM WAT, only CD8^+^ T cells exhibited increased Ki67 expression during acute infection, and only in lean animals.

The effects of SIV infection and ART on T cell memory subsets are shown in Supplemental Figure 3. Naive CD4^+^ T cell frequencies were increased in both lean and obese animals after acute infection in whole blood, while the frequency of naive CD8^+^ T cells decreased in both groups in SC WAT (Supplemental Figure 3A). The frequencies of CD4^+^ central memory (Tcm) T cells decreased in both groups after initial infection in whole blood and OM WAT, but not in SC WAT, but CD8^+^ Tcm frequency increased only in OM WAT after initial infection (Supplemental Figure 3B). Finally, effector memory (Tem) CD4^+^ T cell frequencies decreased in whole blood but decreased in OM WAT only in lean animals (Supplemental Figure 3C). The frequency of CD8^+^ Tem cells also decreased in OM WAT, but only in lean animals. Thus, changes in memory subsets differed between whole blood and different WAT depots as well as between the lean and obese groups.

Since WAT-resident macrophages and their activation/polarization state is thought to be associated with WAT inflammation in obesity, we also examined macrophage activation status in OM and SC WAT as shown in Supplemental Figure 4. iNOS^–^/CD163^+^ antiinflammatory, alternatively activated ‘M2” macrophages as a percent of CD45^+^ cells were predominant over iNOS^+^/CD163^–^ inflammatory, classically activated “M1” macrophages at baseline in both lean and obese animals. The frequency of M1 macrophages decreased after long-term ART in SC WAT, while the frequency of M2 macrophages decreased after long-term ART in OM WAT, but these changes were only significant in the lean group.

SIV-specific T cell and humoral responses were assessed by ELISpot and Env-specific binding antibody assays, respectively. As shown in Supplemental Figure 5A, there were no significant differences in the magnitude or breadth of longitudinal SIV-specific T cell responses between lean and obese animals. As shown in Supplemental Figure 5B, levels of anti-SIVmac239 gp140 antibodies increased rapidly following infection, peaking at 1 month of ART (8 weeks postinfection), and remained elevated for the remainder of the study period. There was no significant difference between the lean and obese groups.

### Long-term ART alters adipocyte size and pericellular extracellular matrix thickness in OM but not SC WAT.

**Figure 4:**
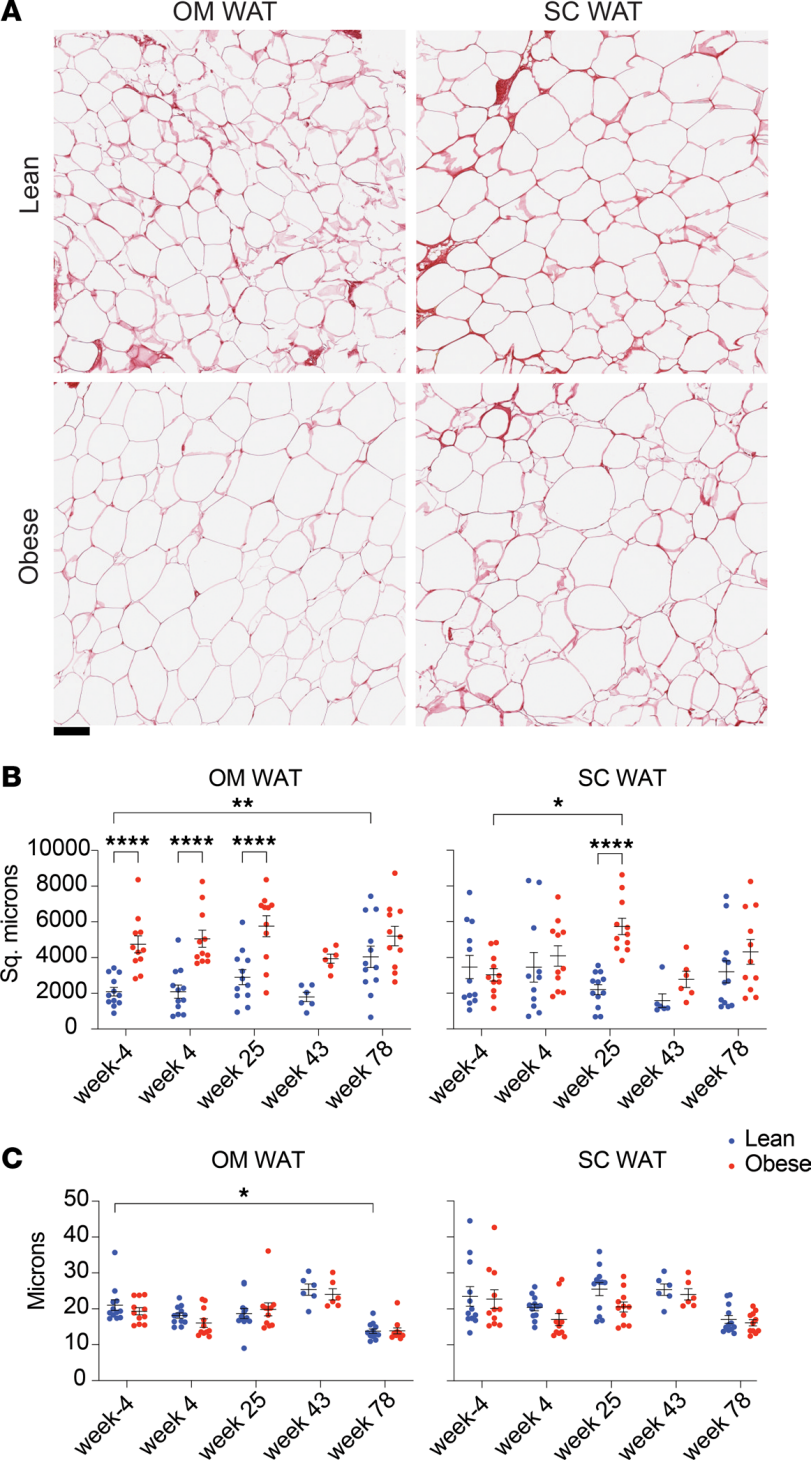
Effect of SIV infection and long-term ART on adipocyte size and pericellular extracellular matrix thickness. (**A**) Representative images of OM and SC WAT from lean and obese animals stained with picosirius red; scale bar: 100 μm. (**B**) Average adipocyte size in individual lean and obese animals over the study time course. (**C**) Average interadipocyte/pericellular extracellular matrix thickness in lean and obese animals over the study time course. Data points for lean animals are in blue and those for obese animals are in red. Significance was determined by mixed-effects analysis with Tukey’s multiple comparison test. *P < 0.05; **P < 0.01; ****P < 0.0001. All data are means ± SEM.

We also evaluated the effect of SIV infection and subsequent ART on adipocyte size and pericellular extracellular matrix (ECM) thickness, which reflects collagen deposition and potential fibrosis. Figure 4A shows representative images of OM and SC WAT from lean and obese animals at baseline, illustrating the increased adipocyte size in obese OM WAT. Figure 4B shows the quantitation of average and individual animal average adipocyte size in OM and SC WAT in lean and obese animals at baseline and over the course of infection and ART. In OM WAT, adipocytes in the obese group were significantly larger than in the lean group at baseline and through infection and early ART, but this difference disappeared by week 78 due to a significant increase in adipocyte size in the lean group. In contrast, there was no difference in SC adipocyte size between the lean and obese groups, other than a transient increase in size in the obese group at week 25. We also analyzed interadipocyte Picrosirius red staining of ECM thickness as a potential marker of pericellular fibrosis, which has been proposed as a more specific indicator of decreased adipocyte expandability and metabolic dysfunction (59–62). As shown in Figure 4C, this parameter was similar in both lean and obese groups in both WAT depots throughout the study, except for a decrease at week 78 in lean OM WAT.

### Differences in body composition parameters between lean and obese animals were largely unchanged by SIV and ART.

DEXA scanning revealed that the greater initial percent body weight loss shown in the obese group in Figure 1B was predominately due to a greater loss of fat mass in this group (Supplemental Figure 6, A–C). As shown in Supplemental Figure 6, D–F, the obese group had significantly higher levels of lean mass, bone mass, and BMD compared with the lean group. However, these parameters did not change significantly over the time course in either group. Thus, in each case, baseline differences between the lean and obese groups were largely maintained.

### Measures of glucose homeostasis improve in both lean and obese animals following long-term ART.

At baseline, the obese group exhibited relative hyperglycemia compared with the lean group in spite of elevated insulin and C-peptide secretion assessed by ivGTTs (Table 1 values for GAUC, IAUC, and CAUC and Supplemental Figure 7, A–C; individual baseline values shown in inserts), reflecting the prediabetic phenotype of the obese group. However, these parameters responded identically over the time course of infection and ART suppression in both groups. Additionally, as shown in Supplemental Figure 7D, the obese group exhibited increased GAUC in an iv insulin tolerance test (ivITT) at baseline (insert), indicating decreased insulin sensitivity in the obese group. However, both groups exhibited improved insulin sensitivity following ART. Supplemental Figure 7E shows that both groups also experienced a decrease in HbA1c during the course of the study. Together, these data suggest that the initial weight stabilization in the lean group and the weight loss in the obese group improved glucose homeostasis, an effect that was maintained even after resumed weight gain.

### SIV and long-term ART do not substantively affect baseline differences in islet function and morphology between lean and obese animals.

Glucose-stimulated insulin secretion (GSIS) was assessed by perifusion of isolated islets obtained at necropsy at 78 weeks postinfection. As shown in Supplemental Figure 8, A and B, basal and total GSIS were greater in islets from obese animals, although first-phase insulin secretion was decreased in islets from obese animals (Supplemental Figure 8C). These findings are consistent with the increased IAUC and CAUC observed in obese animals at baseline (Table 1 and Supplemental Figure 7, B and C), suggesting that the initial phenotypic differences in insulin secretion were maintained throughout the study time course. β and α-cell proportions were assessed by insulin and glucagon IHC. Supplemental Figure 8D shows a representative section stained with insulin and glucagon antibodies. As shown in Supplemental Figure 8, D–F, the proportions of β and α cells and the β/α-cell ratios were not different between the lean and obese groups or in comparison to uninfected control samples.

### Baseline metabolic status affects lipoprotein responses to SIV and ART.

As shown in Supplemental Figure 9A, total cholesterol levels were initially higher in obese animals but dropped in both groups following ART initiation, with levels in the obese group rebounding modestly after prolonged ART. Triglyceride levels were similar in both groups at baseline and modestly increased after prolonged ART. (Supplemental Figure 9B). HDL levels in particular were higher at baseline in the obese group and decreased after ART initiation, while HDL levels in the lean group were not affected by acute infection but also decreased after ART initiation (Supplemental Figure 9C). In contrast, LDL levels were similar in the lean and obese groups at baseline and were subsequently unchanged in the obese group but increased with acute infection and decreased after ART initiation in the lean group (Supplemental Figure 9D).

### Baseline metabolic status influences specific cytokine responses to SIV and ART.

Supplemental Figure 10A shows that the levels of C-reactive protein (CRP), soluble (s)CD14, and LPS binding protein (LBP) increased in both groups following infection and remained elevated throughout the study in both experimental groups. Seven additional factors identified in the Olink assay (Supplemental Figure 10B) exhibited varying patterns of change through the time course of the study, including CCL19, FGF-23, CXCL11, MMP-1, OPG, RANKL/TRANCE, and MCP-1. In most cases, the pattern of change was similar in lean and obese animals, although in some cases the absolute levels varied. Of these, the levels of MCP-1 in particular were also elevated following infection and remained elevated after ART initiation. Thus, several factors were increased in response to infection but were not reversed by ART, consistent with a persistent inflammatory state that is not resolved by ART.

**Figure 5:**
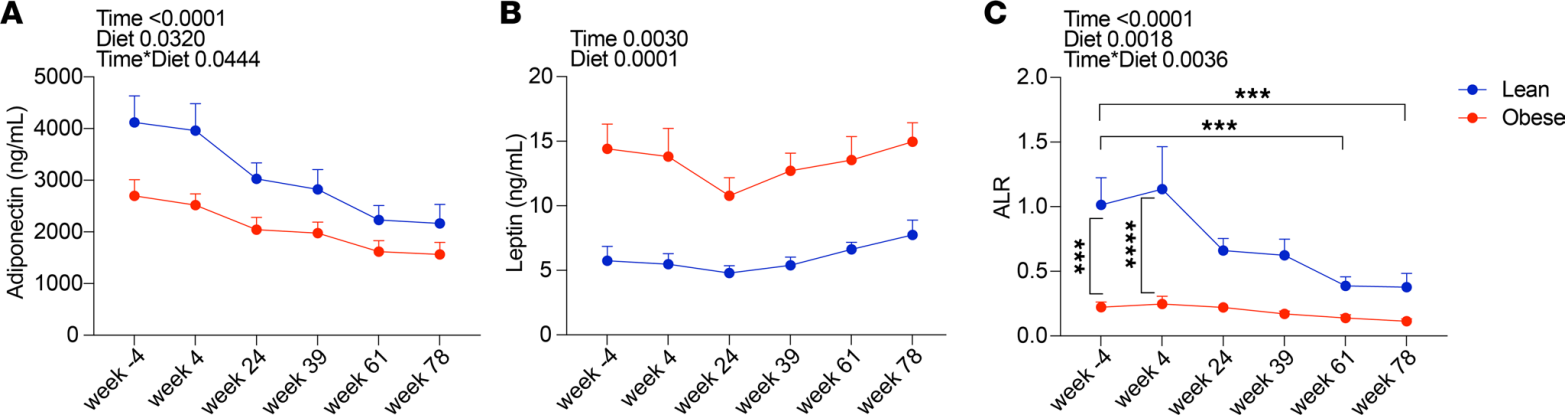
Effect of SIV infection and ART on circulating adiponectin and leptin levels. Total plasma adiponectin (**A**) levels were determined by ELISA and serum leptin (**B**) levels were determined by RIA. *P* values for longitudinal within-group (Time), between-group (Group), and time by group (Time × Group) interaction changes are indicated. (**C**) Adiponectin/leptin ratio (ALR) calculated from data of panels **A** and **B**. Data points for lean animals are in blue and those for obese animals are in red. Significance determined by mixed-effects analysis with Tukey’s multiple comparison test. ***P < 0.001; ****P < 0.0001. All data are means ± SEM.

We also focused on the adipokines adiponectin and leptin that have previously been shown to be affected by SIV and ART (7, 63–66). As shown in Figure 5, A and B, adiponectin levels trended down during SIV infection and ART in both groups, but were higher throughout in the lean group, while the levels of leptin were more constant, although higher in the obese group. As shown in Figure 5C, the adiponectin/leptin ratio (ALR), a marker of obesity, insulin resistance, and WAT inflammation (67, 68), was significantly higher in the lean group at baseline, but was not statistically significantly different from that of the obese group after extended ART. Specifically, the ALR in the lean group declined to under 0.5 by week 61 (56 weeks of ART), a level associated with increased cardiometabolic risk in human patients (67). These data suggest that an effect of long-term ART in lean animals is to convert the ALR phenotype of lean animals to one more characteristic of the obese group. However, we cannot exclude a potential role for viral persistence in altered adipocyte expression of adiponectin in particular, since we did not evaluate adipocyte viral DNA.

### GI inflammation is increased in both lean and obese animals with SIV and attenuated by ART.

Supplemental Figure 11, A–C shows the extent of neutrophil infiltration based on myeloperoxidase staining of the ascending colon following infection and subsequent ART. This parameter is a surrogate marker and response to the breakdown of intestinal epithelial integrity and microbial translocation and mucosal apoptosis and was increased similarly in lean and obese animals following infection but decreased after ART.

## Discussion

We undertook this study to determine, in a systematic and comprehensive fashion, the effects of preexisting metabolic dysfunction, specifically obesity and insulin resistance, on the response to SIV infection and long-term ART in a tractable nonhuman primate model. We sought to exploit the unique advantages of this model, including the use of a clinically relevant ART regimen, a well-defined baseline experimental phenotype of normal and abnormal metabolic status, and the ability to perform an array of experimental assessments, including longitudinal samples of multiple tissues, that are not feasible in clinical studies. To our knowledge, this is the first study in a nonhuman primate model of SIV infection and long-term treatment with a modern ART regimen that also evaluated the effect of infection and ART on multiple tissue responses, including both major WAT depots, in lean and obese animals. Some parameters that were initially different, as expected, between the lean and obese groups at baseline were maintained, while others changed during the course of the study in ways that altered the initial differences between the phenotypes of the lean and obese groups. With respect to the response of the lean and obese groups to infection and ART, many parameters did not differ between the groups, while others were clearly divergent at different points in the course of infection and ART. Thus, the present study generated 2 distinct data sets, one the response of multiple parameters to SIV infection and long-term ART per se, and another, the effects of preexisting obesity/insulin resistance on these same responses.

While some studies have suggested that obesity influences the response to initial SIV infection in the absence of ART (69, 70), we did not observe any significant differences between the groups for peak viremia or the subsequent response to ART with respect to the rate of suppression of plasma viral load. Nor were any differences noted in the suppression of tissue reservoirs represented by decreases in cell-associated viral DNA. These findings are consistent with our assessment of ART tissue concentrations, which did not find any significant differences in the concentrations of ART components in the tissues examined between the groups. We also compared the concentrations of ART components in PBMCs and the adipocyte lipid fraction to address potential accumulation of ART components such as TFV-DP by adipocytes, as previously reported (58). The concentrations of DTG and TVF-DP were orders of magnitude lower in the adipocyte lipid fraction compared with the concentrations of DTG and TFV-DP in PBMCs, and adipocyte lipid fraction concentrations of FTC-TP were undetectable. These data suggest that, at least in this nonhuman primate model, there is not significant differential sequestration of ART components by adipocytes in lean or obese animals. These data are consistent with the lack of appreciable differences in plasma viral RNA and cell-associated viral DNA dynamics between the lean and obese groups and are also in line with a recent report of equivalent ART efficacy in obese and nonobese people with HIV on regimens that included DTG, TFV, and FTC (71). The reported accumulation of TFV-DP by adipocytes in a previous report (70) may reflect the use of in vitro–differentiated adipocytes in the latter study. The persistence of detectable SIV DNA in the SVF fraction in both WAT depots in some animals at necropsy is consistent with the role of WAT as a persistent viral reservoir; however, since we did not measure replication-competent virus, our data do not conclusively show that the WAT SVF fraction, specifically the immune cell component, functions as a long-term reservoir, at least after long-term ART. However, we and others have shown that the majority of virus detected by the cell-associated viral DNA assay we employed comprised replication-competent virus (72–74). Although persistence of SIV would be most likely in WAT SVF immune cells, we cannot rule out the potential presence of persistent virus in mature adipocytes, since they were not assayed.

Although plasma viremia and tissue-associated viral DNA dynamics were similar between the groups, reflecting equal efficacy of ART suppression, there was a significant difference with respect to postinfection weight gain, with the lean group exhibiting a delay in restoration of weight gain until viral control was established, while the obese group exhibited weight loss due to decreased fat mass following infection that was maintained until a rebound after approximately 1 year of ART. This initial weight loss likely contributed to the improvement in the metabolic profile of the obese group as discussed below.

With respect to the effects of SIV and ART suppression on whole blood and WAT immune cell profiles, we observed that SIV infection greatly affected CD4^+^ and CD8^+^ T cell frequencies, particularly in WAT depots, which was more evident in lean animals. Specifically, the frequency of circulating CD4^+^ T cells modestly decreased with SIV infection. However, in both WAT depots, the frequency of CD4^+^ T cells was drastically reduced with acute SIV infection. Although over a year of ART improved the repopulation of CD4^+^ T cells in WAT, the frequencies failed to reach baseline levels, suggesting that SIV infection, even in the presence of ART, has a long-term impact on WAT CD4^+^ T cells. CD8^+^ T cells, on the other hand, accumulated in WAT after infection and in the presence of ART, concordant with increased expression of the activation and tissue retention marker CD69, likely attributed to WAT inflammation induced by SIV infection. This effect was more significant in lean animals. Damouche et al. (28) previously reported, in cynomolgus macaques chronically (15 months) infected with SIV in the absence of ART, modest (approximately 50%) decreases in CD4^+^ T cell frequencies in WAT depots and increases in WAT CD8^+^ T cell frequencies, without drastic changes in the proliferative marker Ki67. In contrast, we observed a significant increase in Ki67 expression in CD4^+^ and CD8^+^ T cells in both OM and SC WAT with SIV infection. These discrepancies are likely due to the timing of infection, where Damouche et al. analyzed WAT in chronically infected animals, whereas we detected changes in proliferation in acute infection. Although there was no impact of obesity on the humoral response, we did observe an impairment, albeit not statistically significant, in early virus-specific T cell responses in obese animals compared with lean animals. This diminished T cell response may be a result of chronic inflammation associated with obesity and warrants further exploration. We also observed that the majority of WAT CD4^+^ and CD8^+^ T cells were central or effector memory cells throughout the study time course, similar to what was reported by Coutrier et al. (26) and Damouche et al. (28) in chronic SIV infection. The differences we observed between WAT T cell subtypes in whole blood and WAT presumably reflect the emerging picture of the distinct nature of WAT immune cells (75).

We also characterized WAT macrophage profiles and found that antiinflammatory M2 macrophages predominated over inflammatory M1 macrophages in both lean and obese groups throughout the study time course, although the frequency of M2 macrophages decreased in OM WAT and the frequency of M1 macrophages decreased in SC WAT after extended ART, but only in lean animals. These results suggest that WAT-resident macrophage polarization is not significantly affected by SIV and initial ART treatment. It is also important to note that these relative frequencies were similar in lean and obese animals, which differs from the original M1/M2 paradigm in rodent models of obesity, in which M1 macrophage frequency increases and M2 macrophage frequency decreases during the development of obesity. We acknowledge that the characterization of WAT macrophages as M1 or M2 is an oversimplification (76), but our data do show that the preponderance of antiinflammatory M2 macrophages is not affected by baseline obesity and insulin resistance or by SIV infection and ART. Damouche et al. (28) previously reported that WAT M2 macrophages were predominant in control animals, but that WAT M1 macrophage frequencies were increased and WAT M2 macrophage frequencies were decreased in chronic SIV infection. In our studies, ART presumably prevented the changes in WAT macrophage polarization described by Damouche et al.

In light of the role of WAT as an important SIV and HIV reservoir, we also assessed the effect of SIV and ART on adipocyte morphology, specifically adipocyte cell size and the thickness of the interadipocyte ECM as a surrogate marker for potential pericellular fibrosis. Obese animals had larger adipocytes at baseline in OM but not SC WAT. After long-term ART, the size of OM adipocytes in lean animals approached that of obese animals. This differential response may be associated with the decrease in plasma adiponectin specifically in lean animals discussed below. In contrast, Damouche et al. (28) reported an increase in adipocyte size (decreased density) in both WAT depots in SIV-infected animals after 15 months of SIV infection without ART, suggesting that ART does not prevent an increase in OM adipocyte size, but may prevent an increase in SC adipocyte size.

We did not observe any effect of SIV or ART on WAT ECM thickness in lean or obese animals, other than a small decrease in OM WAT in lean animals at week 78. In contrast, Gorwood et al. (77) and Ayissi et al. (78) reported increased collagen staining in OM and SC WAT from chronically SIV-infected or SIV-infected/ART-treated animals, respectively. The discrepancy between those results and ours may be due to chronic infection versus ART in the first case and the assessment of total ECM staining in the second, whereas our analysis was restricted to pericellular ECM thickness, which is more associated with adipocyte dysfunction (58–61).

Given the importance of WAT as both a viral reservoir and a contributor to overall metabolic status, we were particularly interested in the responses of the major adipokines adiponectin and leptin to SIV and ART. The principal effect observed was a decrease in the adiponectin/leptin ratio, a marker of cardiometabolic risk, in lean animals following initiation of ART. This effect was mainly driven by a decrease in adiponectin in the lean group. Thus, ART altered the relative expression of these factors in the lean group to a pattern characteristic of the obese group. Couturier et al. (27) reported increased circulating leptin and unchanged adiponectin in chronic SIV-infected animals in the absence of ART, while Offor et al. (63), Hulgan et al. (7), and Hikasa et al. (65) described ART-associated decreases in adiponectin and/or ALR in persons with HIV, consistent with our results. GLP-1 receptor agonists such as exendin-4, exenatide, liraglutide, and tirzepatide have been reported to increase adiponectin levels (79–82), and semaglutide has been shown to reduce cardiometabolic risk (83). These data, in conjunction with our finding that the ALR is decreased in lean SIV-infected, ART-treated animals, suggest that GLP-1 receptor agonists may attenuate metabolic comorbidity in nonobese as well as obese PLWH.

Since the primary focus of our study was on the potential effects of ART on various aspects of metabolic disease and the potential additional burden of preexisting obesity and insulin resistance, we also monitored an array of additional metabolic parameters, including body composition indices, systemic glucose metabolism, islet function and morphology, lipid profiles, cytokine profiles, and colon inflammation. For the most part, the response of these parameters to SIV infection and ART suppression did not appreciably differ between lean and obese animals, with baseline differences, where present, being largely maintained throughout the study time course. Some parameters, such as insulin resistance, improved after ART, but were evident in both lean and obese animals due to weight stabilization in the former and weight loss in the latter. Other parameters, such as CRP, sCD14, and LBP levels, increased with infection and remained elevated after ART, representing persistent SIV-induced inflammation in both groups, while colon neutrophil invasion increased with infection but resolved with ART, again in both groups. Overall, the magnitude and extent of new or exacerbated metabolic dysfunction was modest, with effects of SIV and ART and the additional effect of obesity potentially obscured by preferential weight loss in the obese group and the duration of the study. Since the lean group did not exhibit significant weight gain and the obese group did not exhibit significant weight regain until relatively late in the study, adverse effects of ART-induced metabolic function due to increased BW may require a longer study period. Another potential factor was our use of TDF in the standard SIV ART regimen rather than tenofovir alafenamide, since the latter is associated with greater weight gain and metabolic comorbidities than TDF (84–87). A second aspect of the standard nonhuman primate ART regimen that may have reduced the extent of metabolic comorbidities is the potential antiinflammasome activity of cytosine analog nucleoside reverse transcriptase inhibitors such as lamivudine and FTC (88, 89), particularly with respect to WAT function and overall metabolic status (90, 91).

The use of only male macaques is also a limitation of the study. Since women are still underrepresented in clinical studies of HIV and ART (92), and there are differences in the spectrum and frequency of ART-associated comorbidities (93–95), similar studies in the macaque model using females are warranted. An additional limitation is the use of obese animals whose weight gain was induced by consumption of an obesogenic Western-style diet (WSD). We consider the differences that we observed between the lean and obese groups to be primarily due to obesity and/or associated IR, but we cannot exclude the possibility that the differences seen reflect, at least in part, an effect of the WSD independent of obesity. A final limitation of this study is the initiation of ART at 5 weeks after infection, soon after peak viremia. This may make our conclusions primarily translatable to acutely treated patients.

In spite of these limitations, the present study represents what is, to our knowledge, the first comprehensive combined viral, immunological, and metabolic description of the nonhuman primate model of SIV infection and a modern ART regimen. We have provided a solid foundation for future use of this model to investigate more nuanced effects of preexisting metabolic disease on metabolic outcomes such as metabolomic and lipidomic profiles as well as investigation of adjunct therapeutic approaches to complement ART and reduce metabolic comorbidities, including established drugs such as metformin or newer candidates such as glucagon-like peptide receptor-1 receptor agonists.

## Methods

### Sex as a biological variable.

This study utilized adult male rhesus macaques. Limited availability of female macaques for research and budget constraints precluded the use of both sexes, although examination of the effects of obesity and insulin resistance on the response to SIV and ART in female animals would be an important future goal.

### Animals.

This study employed a total of 22 SIV-naive adult male rhesus macaques negative for the protective alleles Mamu-B*17 and Mamu-B*08 that are associated with spontaneous elite control of SIV (96). Study animals were grouped into 2 cohorts studied consecutively (Figure 1A). The first cohort consisted of 6 lean, metabolically healthy animals and 6 obese, insulin-resistant animals; however, 1 animal in the obese group was excluded from the study due to the development of diabetes early in the study. The second cohort consisted of 6 lean and 5 obese animals. Lean animals were fed a control diet with 15% calories derived from fat (Purina 5000/5052; LabDiet). Obese animals were selected from the ONPRC Obese Nonhuman Primate Resource, which maintains a colony of animals fed a WSD containing 36% calories from fat (Purina 5LOP, LabDiet). Obese animals chosen for this study had been on the WSD for at least a year and exhibited a consistent obese/insulin-resistant phenotype. Animals were typically pair-housed in the same treatment group and maintained on a 07:00–19:00 light cycle with ad libitum access to water. Individual enrichment devices were provided on each cage and animals were given weekly access to additional enrichment activities (radio, TV, etc.). All sedations for procedures were induced with ketamine HCl (5–15 mg/kg) (Covetrus) or a tiletamine HCl and zolazepam HCL mixture (3–5 mg/kg) (Telazol; Zoetis Inc.) unless otherwise noted.

### SIV infection.

After completion of baseline assessments, all animals were infected i.v. with 10,000 IU of barcoded SIVmac239M (97, 98). Blood draws were taken weekly thereafter for monitoring of plasma viral load.

### ART regimen.

At 5 weeks postinfection, an ART regimen consisting of DTG, TDF, and FTC, coformulated as a once-daily injection (99, 100), was initiated and continued for a subsequent 16 months.

### Determination of plasma SIV RNA and cell-associated viral DNA.

Plasma SIV RNA levels were determined by quantitative RT-PCR as previously described (101). The LOQ for this assay is 15 copies of viral RNA per mL of plasma. Cell-associated SIV DNA levels from tissues were determined as previously described (102, 103). The LOQ for this assay is 26 copies of viral DNA per million cells.

### Analysis of tissue ART drug concentrations.

Intracellular levels of DTG, TFV-DP, and FTC-TP in PBMCs, mesenteric lymph node, inguinal lymph node, spleen, OM and SC WAT SVF cells, and the lipid fraction of the adipocyte component of OM and SC WAT were determined as previously described (70). Preparation of the WAT adipocyte lipid fraction for determination of adipocyte ART drug levels is described below. Calculation of the volume of PMBCs/mL used in the data shown in Figure 3B was based on the average PBMC volume reported for PLWH (104).

### PBMC and SVF immune profiling by flow cytometry.

Lymphocyte frequencies were monitored by whole blood staining as previously described (74). Briefly, EDTA-treated whole blood (50–100 μL) was washed twice with 1 × PBS and then stained for surface markers and viability for 30 minutes at room temperature (RT). After staining, whole blood was resuspended in 1 mL 1 × FACS Lysing Solution (Becton Dickinson) to lyse red blood cells and fix remaining cells, incubated for 8 minutes, and washed 3 times with 1 × PBS supplemented with 10% FCS (FACS buffer). After the last wash, 500 mL FACS perm buffer (1 × Becton Dickinson FACS Lysing Solution + 0.05% Tween-20) was added and incubated for 10 minutes at RT. Cells were then washed 3 times with FACS buffer. For Ki67 assessment, fixed and permeabilized cells were stained for 45 minutes at RT. Following intracellular staining, samples were washed twice with FACS buffer and data was acquired on an LSR II flow analyzer (Becton Dickinson). Flow cytometric data were analyzed using FlowJo, version 10 (TreeStar). Antibodies used are listed in Supplemental Table 1 and gating strategies are demonstrated in Supplemental Figure 11. Absolute lymphocyte counts were calculated by multiplying whole blood staining frequencies within the live CD45^+^ gate by the white blood cell count, as determined by complete blood count performed on an additional aliquot of EDTA-treated whole blood.

### Isolation of WAT adipocyte lipid fraction and SVF.

WAT biopsy or necropsy samples were processed as previously described (105). In brief, 1–5 grams OM-WAT and SC-WAT were collected and promptly transported to the laboratory in ice-cold FACS buffer (DPBS, Thermo Fisher Scientific, supplemented with 0.5% BSA (Sigma-Aldrich) and 2 mM EDTA (Sigma-Aldrich). Upon arrival at the laboratory, WAT was minced and subjected to a 30–40 minute digestion with continuous mixing in a collagenase solution prepared by combining 120 mg of collagenase type-II (Gibco, 17101-015), 1.4 g of BSA (Sigma-Aldrich), DPBS (to 40 mL), and 80 μL of 1 M CaCl_2_. Following digestion, 40 mL of ice-cold FACS buffer was added and the resulting cell suspension was filtered through a 100-μm cell strainer. The cell suspension was then centrifuged at 400*g* for 10 minutes at 4°C, and the supernatant, along with the top lipid fraction, was discarded in the case of biopsy samples. For necropsy samples, the top lipid fraction was collected and its volume was recorded. To the SVF pellet, 10 mL of red blood cell lysis buffer was added, followed by an 8 minute incubation at room temperature. The SVF cell pellet was then washed twice with ice-cold FACS buffer through centrifugation at 400*g* for 10 minutes at 4°C. Finally, the pellet was resuspended in 1 mL of FACS buffer on ice for subsequent cell counting and flow cytometry analysis. For determination of ART drug concentrations in the adipocyte lipid fraction, the lipid fraction was diluted with 2 volumes of methanol and kept at –20°C until analysis.

### WAT histology and determination of adipocyte size and ECM thickness.

WAT histology was performed as described (105). Briefly, 200–500 mg fragments of SC-WAT and OM-WAT were collected at necropsy and fixed in zinc formalin (Thermo Fisher Scientific) at 4°C for 48 hours. Samples were transferred to 70% (v/v) ethanol for 5 days, embedded in Paraplast wax (Leica), and 5 μm sections were prepared using a micron rotary microtome. Slides were stained with Picrosirius red Stain Kit (Abcam) according to the manufacturer’s instructions. Images representing tissue segments of approximately 5–10 mm in size were acquired using an Aperio AT2 System slide scanner (Leica Biosystems). Adipocyte size and pericellular ECM thickness were determined as described (106).

### Statistics.

Statistical analyses were performed using GraphPad Prism (GraphPad Software). Longitudinal data were assessed using either 2-way ANOVA with Šidák’s multiple comparisons test or mixed-effect analysis with Šidák’s multiple comparisons test. Individual comparisons between the lean and obese groups at baseline were assessed using unpaired 2-tailed *t* tests. AUC was calculated starting at *T* = 0. A *P* value < 0.05 was considered significant.

### Study approval.

All studies were approved by the Oregon National Primate Research Center IACUC and were conducted in accordance with the US Public Health Service’s Policy on Humane Care and Use of Laboratory Animals.

### Data availability.

All raw data associated with data presented in the manuscript are included in the Supporting Data Values file.

## Author contributions

PK, JBS, and CTR designed the study. GMW, KAS, DT, MK, LB, SRL, HB, CZ, MS, CM, HH, UA, CP, JMH, AM, MH, JR, OV, LW, and CVF conducted experiments and acquired data. GMW, KAS, PK, JBS, CTR, OV, LG, CVF, and TTB interpreted results. PK, JS, and CTR wrote the manuscript and are cosenior authors. All authors reviewed and edited the manuscript. GMW and KAS are cofirst authors for this study. Both had significant roles in study management and assistance with data organization, interpretation, and manuscript preparation, but the contribution of GMW was more substantial with regards to duration of effort and scope of data responsible for.

## Supplementary Material

Supplemental data

Supporting data values

## Figures and Tables

**Table 1:**
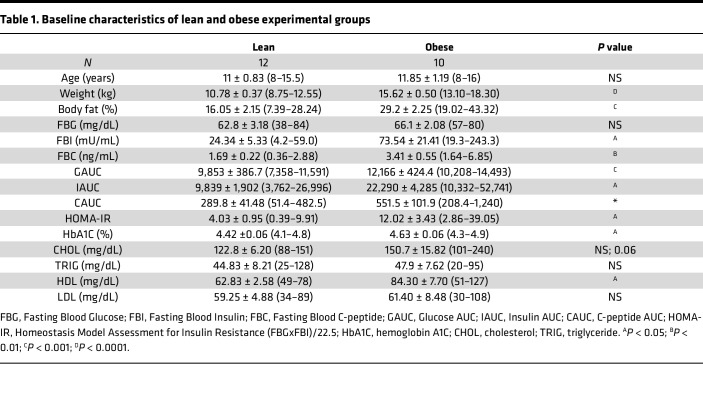
Baseline characteristics of lean and obese experimental groups

## Version 1 08/08/2024

In-Press Preview

## Version 2 09/24/2024

Electronic publication
