# Supplementary material for: Effect of metabolic status on response to SIV infection and antiretroviral therapy in nonhuman primates
Source: JCI Insight. 2024 Aug 8;9(18):e181968. doi: 10.1172/jci.insight.181968 (PMC11457846; doi:10.1172/jci.insight.181968)
Supplement: Supplemental data [file jciinsight-9-181968-s168.pdf]

Effect of metabolic status on response to SIV infection and antiretroviral therapy in nonhuman primates

Gabriela M. Webb[1], Kristin Sauter[2], Diana Takahashi[2], Melissa Kirigiti[2], Lindsay Bader[2], Sarah R. Lindsley[2], Hannah Blomenkamp[2], Cicely Zaro[2], Molly Shallman[2], Casey McGuire[2], Heather Hofmeister[2], Uriel Avila[2], Cleiton Pessoa[1], Joseph M. Hwang[1], Allyson McCullen[1], Matthew Humkey[1], Jason Reed[1], Lina Gao[3], Lee Winchester[4], Courtney V. Fletcher[4], Oleg Varlamov[2], Todd T. Brown[5], Jonah B. Sacha[1], Paul Kievit[2], and Charles T. Roberts[2,6]

[1]Division of Pathobiology and Immunology, Oregon National Primate Research Center (ONPRC), Beaverton, OR, USA; [2]Division of Metabolic Health and Disease, ONPRC; [3]Knight Cancer Institute, Oregon Health & Science University, Portland, OR, USA; [4]Antiviral Pharmacology Laboratory, Center for Drug Discovery, University of Nebraska Medical Center, Omaha, NE, USA; [5]Division of Endocrinology, Diabetes, and Metabolism, Johns Hopkins University, Baltimore, MD, USA; [6]Division of Reproductive and Developmental Sciences, ONPRC.

This PDF file includes:
Supplemental Table 1
Supplemental figures 1-12
Supplemental Methods
Supplemental References 1-4

Supplemental Table

Table S1. **List of antibodies used for flow cytometry and immunohistochemistry in this study.**

Supplemental table 1. **Antibodies used for flow cytometry and histochemistry in this study.**				
Antibodies used for flow cytometry.				
**Antibody target**	**Clone**	**Host species**	**Source**	**Catalog #**
CD4	L200	Mouse	BD Biosciences	564107
CD8	SK1	Mouse	BioLegend	344714
CD45	D058-1283	Mouse	BD Biosciences	563861
CD28	CD28.2	Mouse	BD Biosciences	560684
CD3	SP34-2	Mouse	BD Biosciences	558124
CD95	DX2	Mouse	BioLegend	305612
CD38	OKT10	Mouse	Nonhuman primate reagent resource	PR-3802
HLA-DR	G46-6	Mouse	BD Biosciences	560743
CD69	FN50	Mouse	BioLegend	310942
Ki67	B56	Mouse	BD Biosciences	556026
CD45	D058-1283	Mouse	BD Biosciences	561294
HLA-DR	G46-6	Mouse	BD Biosciences	560743
CD3	SP34-2	Mouse	BD Biosciences	557757
CD8	SK1	Mouse	BD Biosciences	561423
CD20	2H7	Mouse	BD Biosciences	561423
CD14	M5E2	Mouse	BioLegend	301837
CD11b	ICRF44	Mouse	BD Biosciences	740965
CD16	3GB	Mouse	BD Biosciences	563785
CD163	GHI/61	Mouse	BioLegend	333606
CD68	KP1	Mouse	Santa Cruz Biotechnology	sc-20060
CD206	19.2	Mouse	BD Biosciences	550889
iNOS	4E5	Mouse	Invitrogen	MA5-17139
Live/Dead ARD	-	-	Life Technologies	34959
Antibodies used for immunohistochemistry.				
**Antibody target**	**Clone**	**Host species**	**Source**	**Catalog #**
Insulin	-	Mouse	Santa Cruz Biotechnology	Sc-377071
Glucagon	-	Guinea pig	Millipore	4031-01F
Myeloperoxidase	-	Rabbit	Agilent	A039829-2

*conjugated in-house using DyLight 405 Conjugation kit

Supplemental Figures

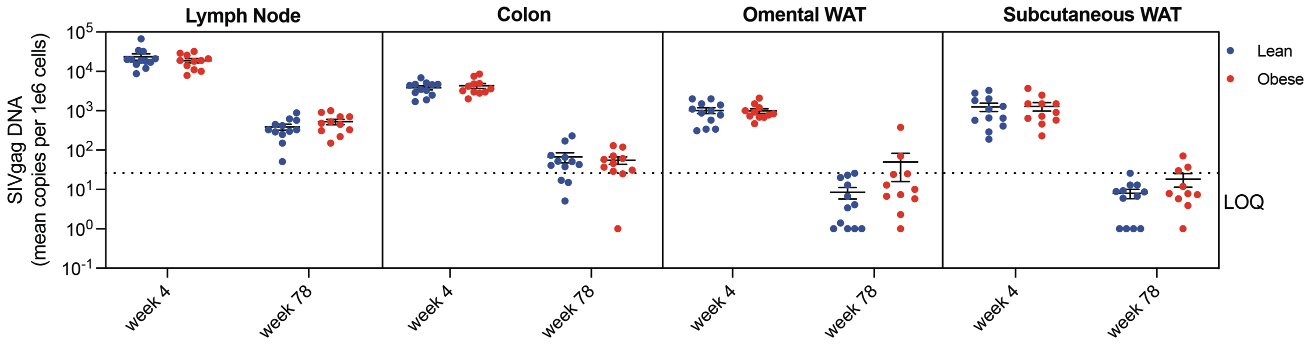

Supplemental Figure 1. **Cell-associated viral loads at peak viremia (week 4 post-infection) and at necropsy (week 78).** Dispersed cells from peripheral lymph node, colon, and the SVF of omental and subcutaneous WAT were analyzed for viral RNA levels as described in Methods. In all figures, datapoints for lean animals are in blue and those for obese animals are in red. All data are means ± SEM.

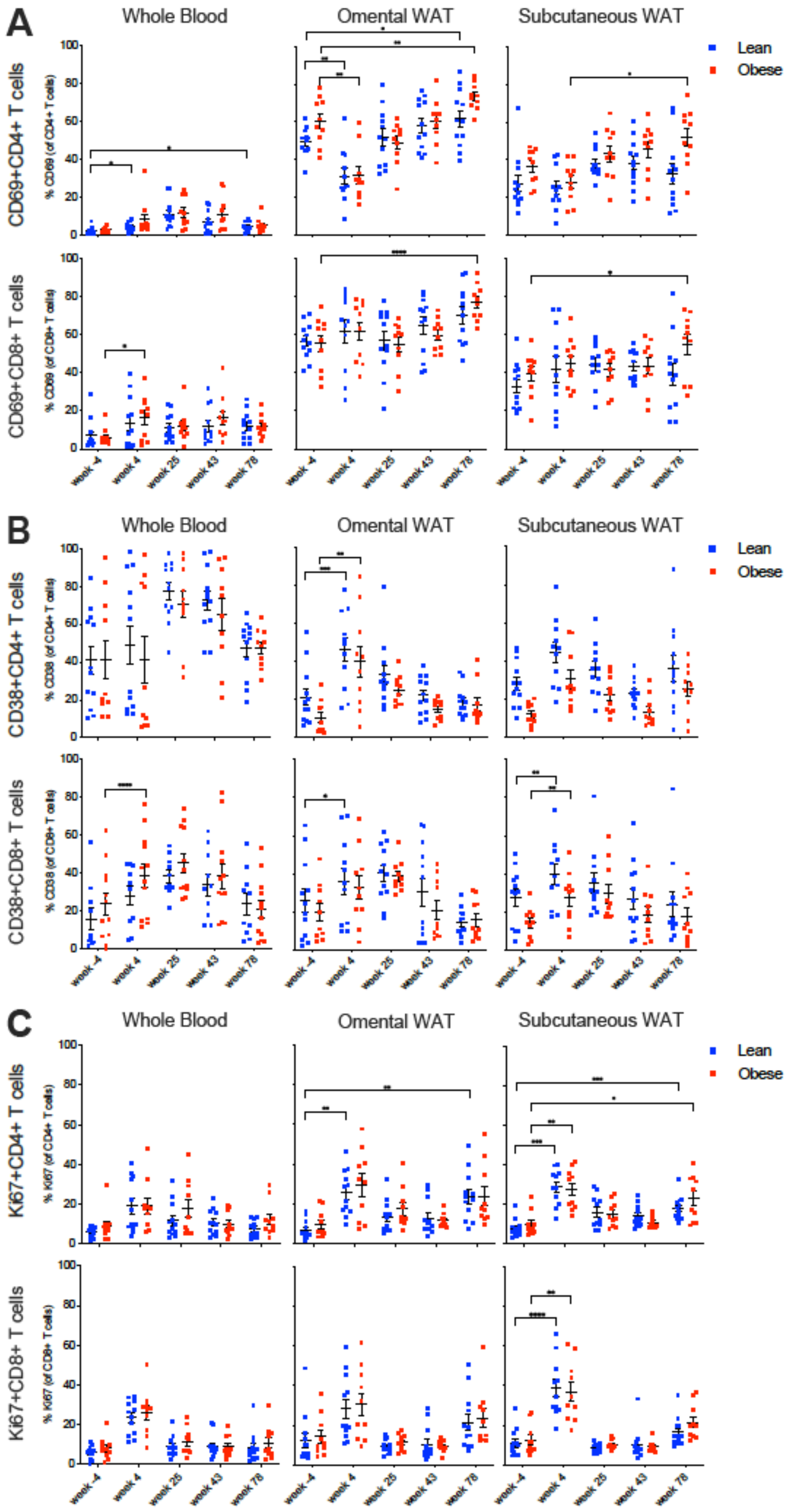

Supplemental Figure 2. **Effect of SIV infection and ART on T cell activation and proliferation markers.** Frequencies of CD69+ (A), CD38+ (B), and Ki67+ (C) CD4+ and CD8+ T-cells in whole blood and omental and subcutaneous WAT determined by flow cytometry. Significance was determined by mixed-effects analysis with Tukey's multiple comparison test. *, $p<0.05$; **, $p<0.01$; ***, $p<0.001$; ****, $p<0.0001$. All data are means ± SEM.

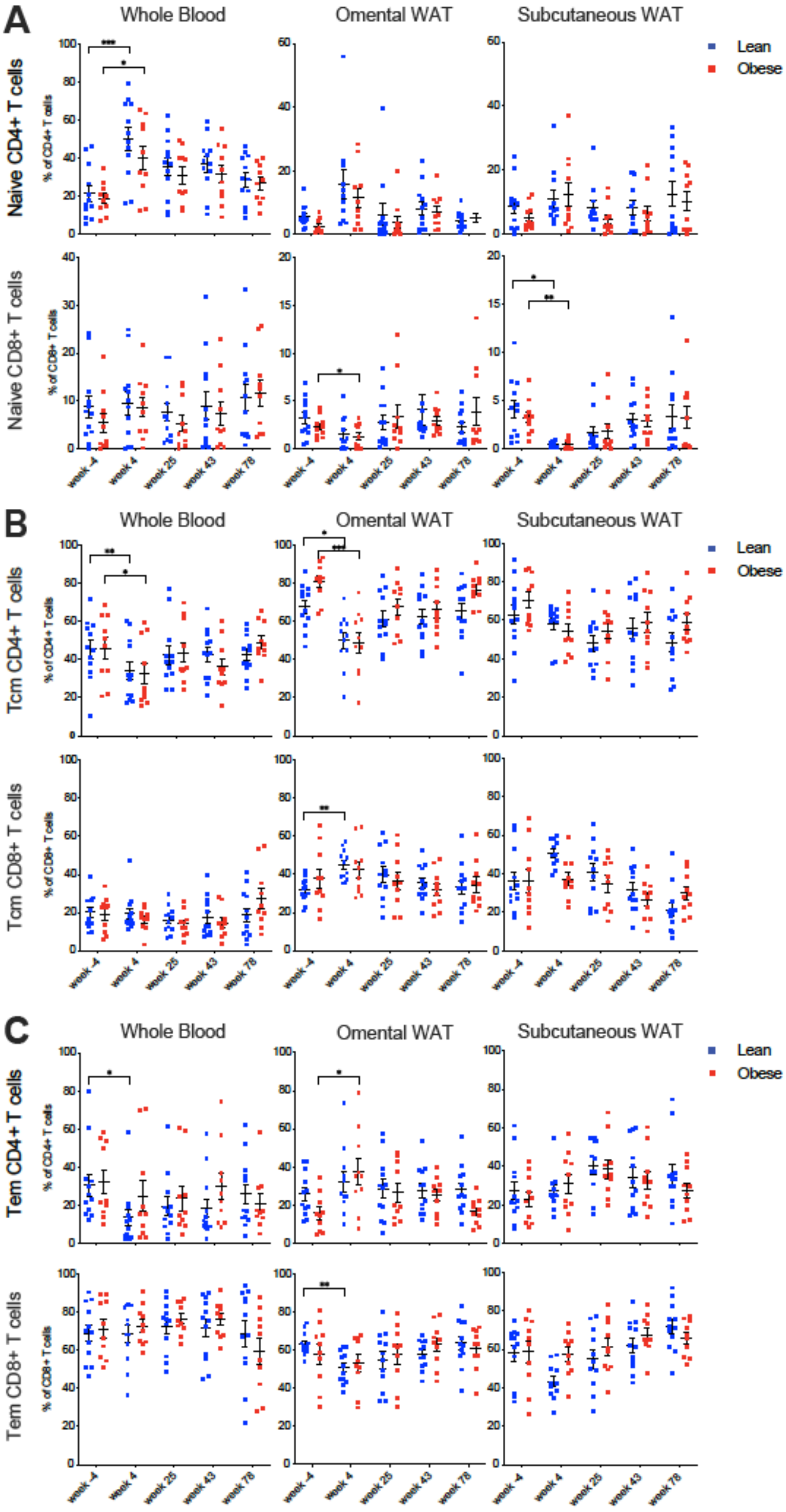

Supplemental Figure 3. **Effect of SIV infection and ART on T cell memory subsets.** Frequencies of naive (A), central memory (Tcm) (B), and effector memory (Tem) (C) CD4+ and CD8+ T-cells in whole blood and omental and subcutaneous WAT determined by flow cytometry. Significance was determined by mixed-effects analysis with Tukey's multiple comparison test. *, $p<0.05$; **, $p<0.01$; ***, $p<0.001$. All data are means ± SEM.

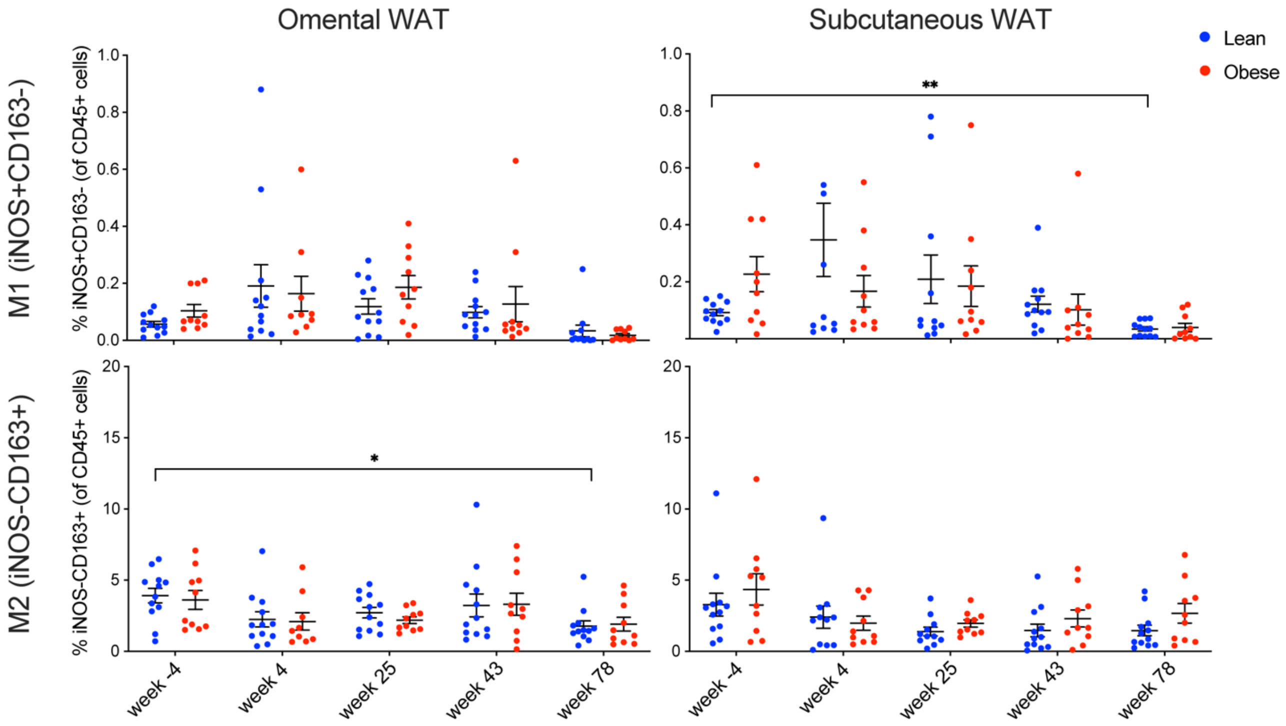

Supplemental Figure 4. **Effect of SIV infection and ART on WAT macrophage polarization.** Frequencies of iNOS-/CD163+ "M2" iNOS+/CD163- "M1" macrophages as a % of CD45+ cells in whole blood and omental and subcutaneous WAT were determined by flow cytometry. Significance was determined by mixed-effects analysis with Tukey's multiple comparison test. *, p<0.05; **, p<0.01. All data are means ± SEM.

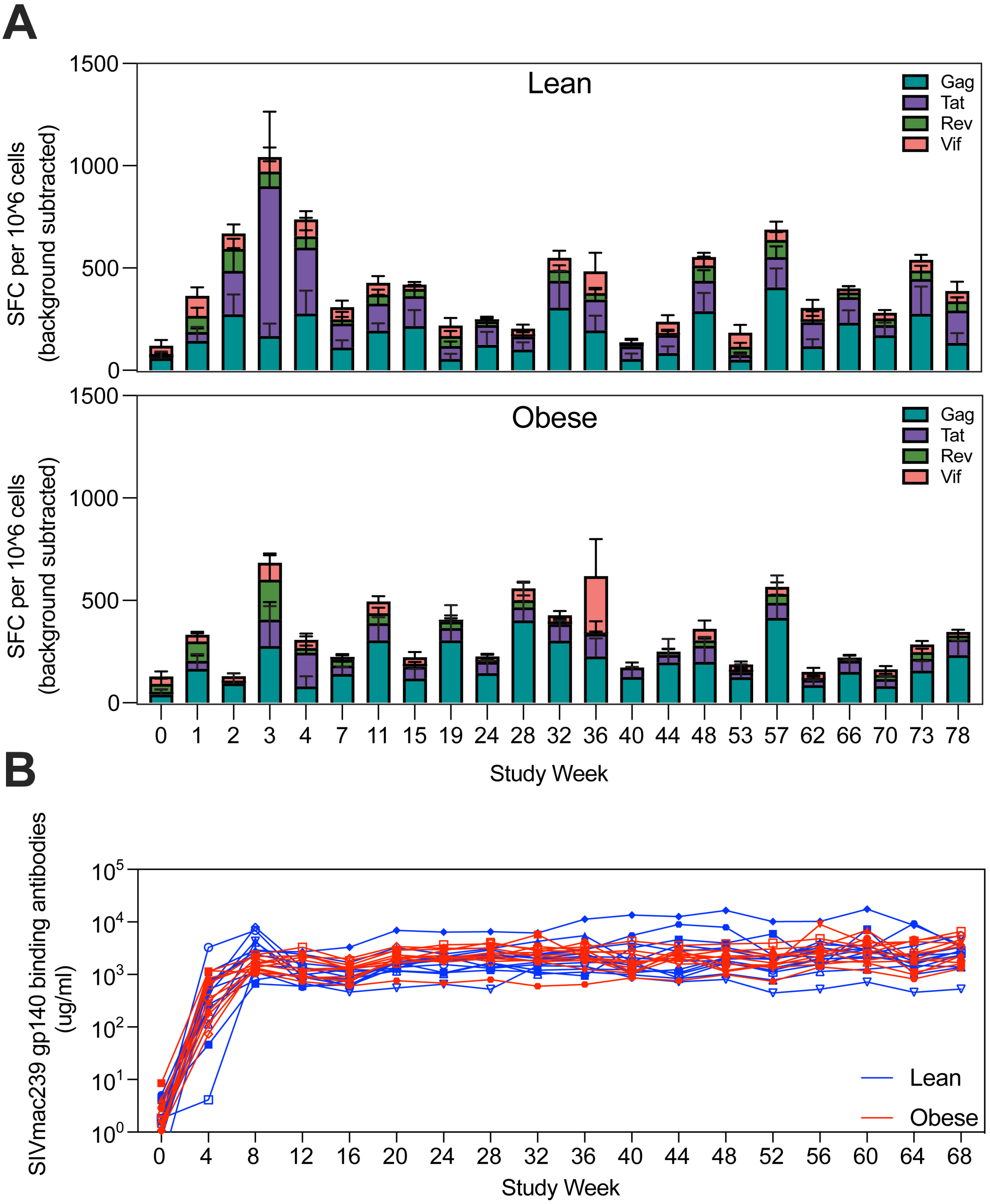

Supplemental Figure 5. **Effect of SIV infection and ART on SIV-specific immune responses.** A. IFN-γ spot-forming cells (SFC) per 10^6 mononuclear cells were quantified by ELISpot upon stimulation with subpools of peptides spanning SIVmac239 Gag, Tat, Rev, and Vif. B. Profile of SIVmac239 gp140-binding antibodies over the course of SIV infection and ART. All data are means ± SEM.

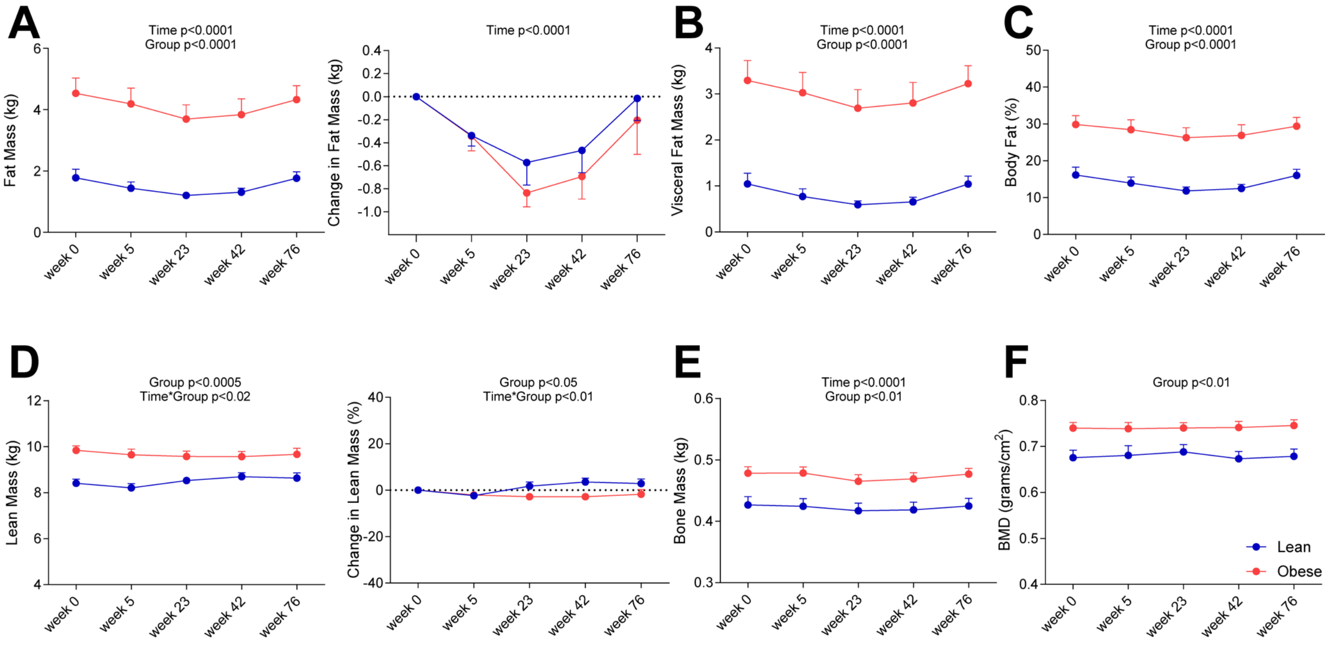

Supplemental Figure 6. **Effect of SIV infection and ART on body morphometric parameters.** Dual-energy X-ray absorptiometry (DEXA) was employed to determine fat mass (A), visceral fat mass (B), body fat % (C), lean mass (D), bone mass (E), and bone mineral density (F). P values for longitudinal within-group (Time), between-group (Group), and time by group (Time*Group) interaction changes are indicated. Significance was determined by two-way ANOVA. All data are means ± SEM.

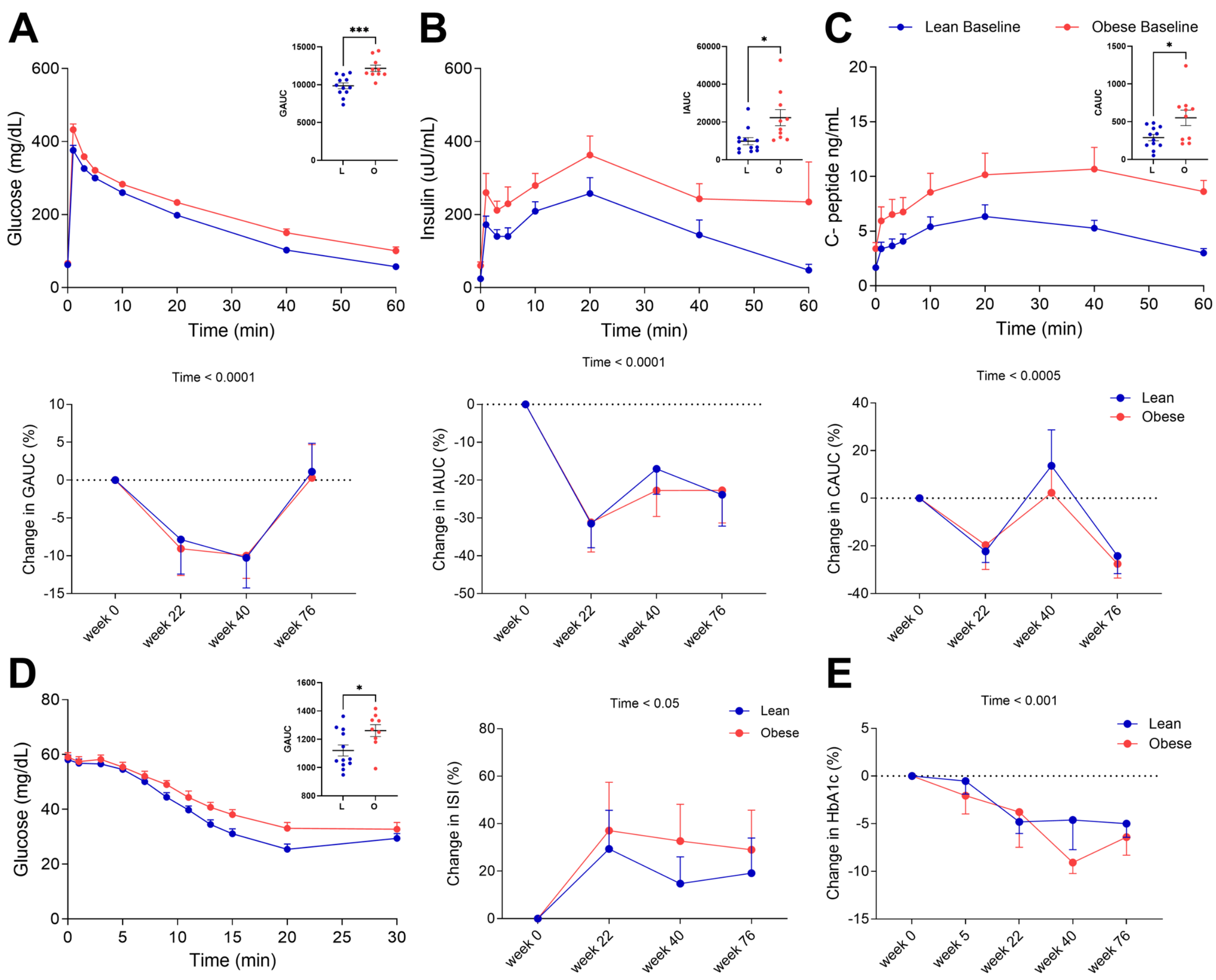

Supplemental Figure 7. **Effect of SIV infection and ART on parameters of glucose homeostasis.** Glucose (A), insulin (B), and C-peptide (C) baseline time course during an ivGTT (individual AUCs shown in inserts) and subsequent longitudinal % change in respective AUCs. D. Glucose profile during a baseline ivITT and subsequent longitudinal % change in ISI. Insert shows individual GAUC values. E. % change in HbA1c over study time course. P values for longitudinal within-group (Time) changes are indicated. Significance in inserts was determined by unpaired t tests. *, p<0.05; ***, p<0.001. All data are means ± SEM.

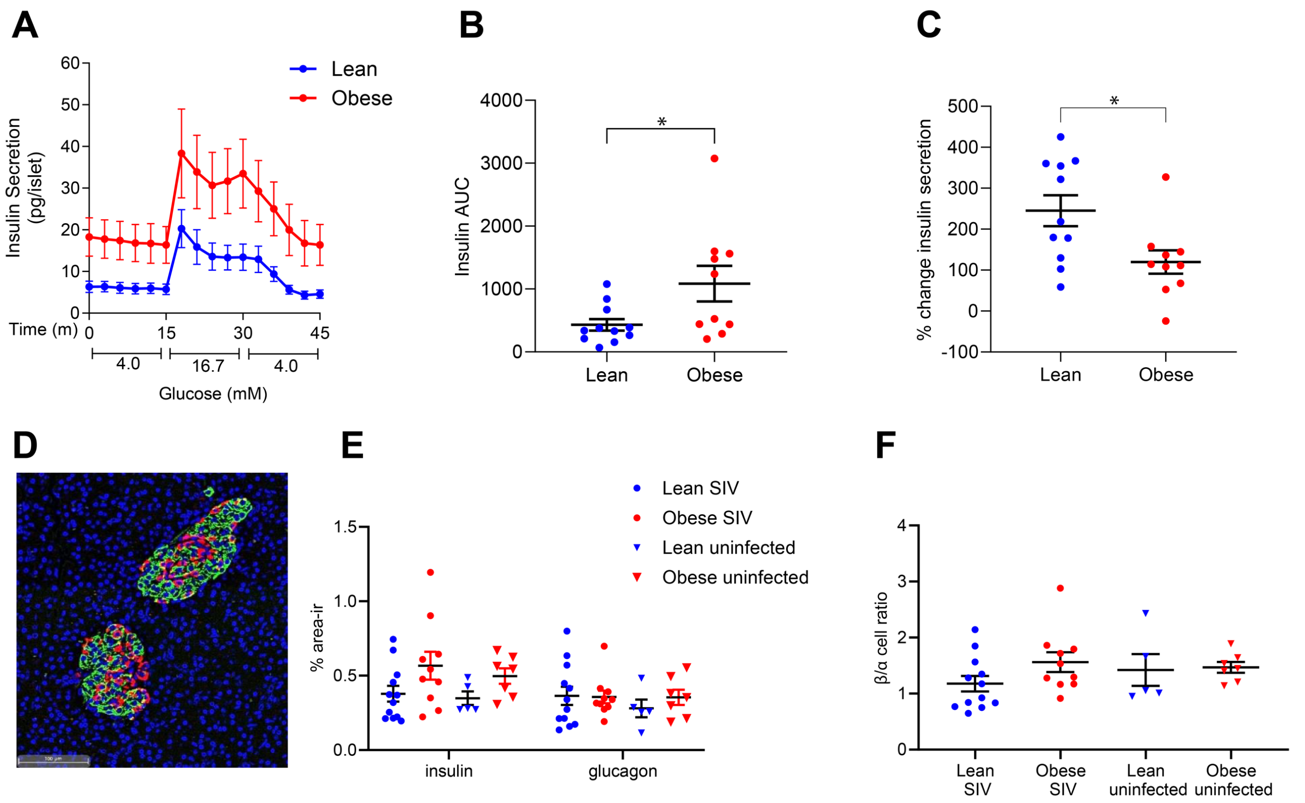

Supplemental Figure 8. **Effect of SIV infection and ART on islet function and morphometry.** A. Glucose-stimulated insulin secretion during perifusion of islets isolated from lean or obese animals. B. Total insulin AUC. C. % change in first-phase insulin secretion (% change during the first 3 minutes of high-glucose challenge). D. Representative image of insulin (green) and glucagon (red) immunohistochemistry in pancreata collected at necropsy, scale bar 100 μm. E. % area of insulin or glucagon immunoreactivity (-ir) in pancreas tissue from lean and obese study animals compared to historical age and BW-matched uninfected controls. F. β/α cell ratio determined from data shown in panel E. Significance was determined by unpaired t tests. *, $p<0.05$. All data are means ± SEM.

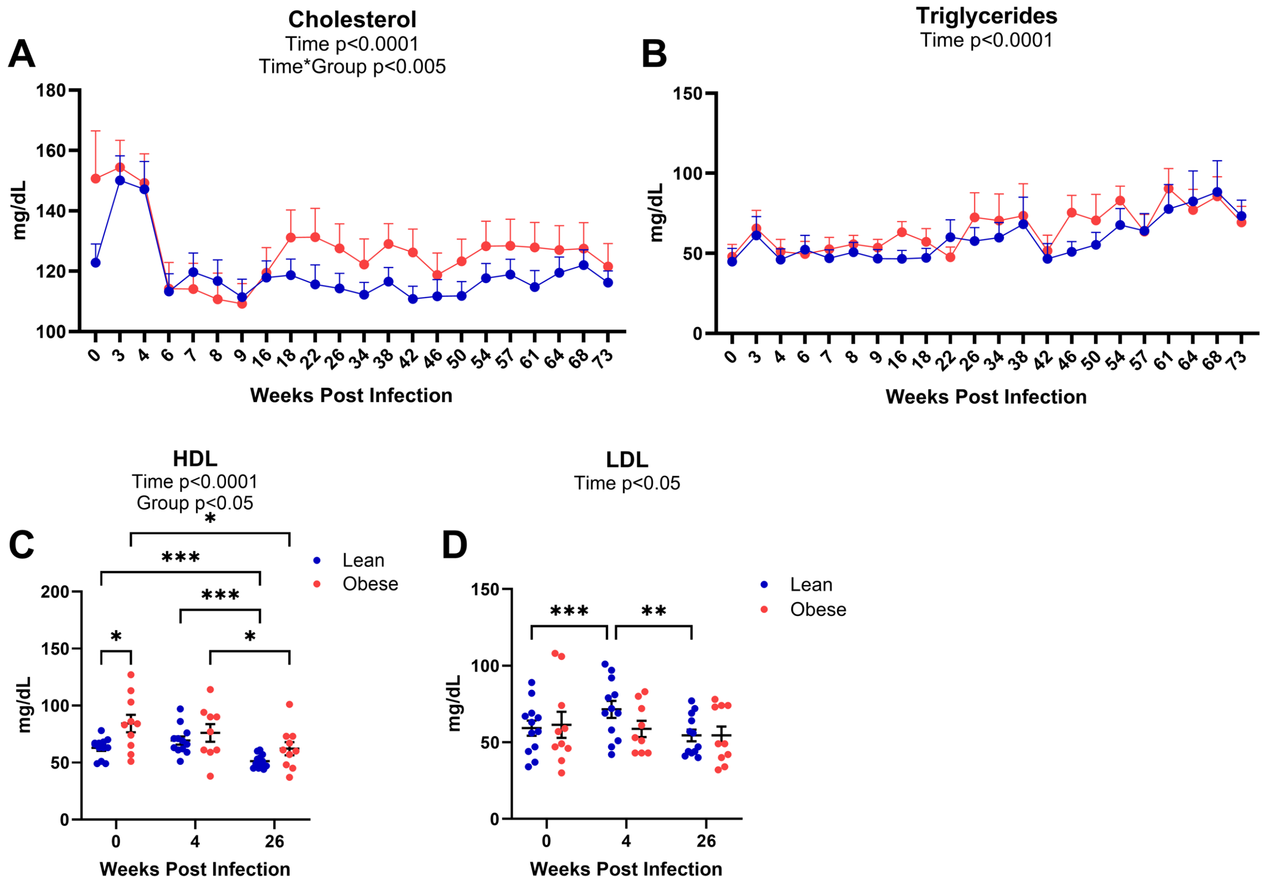

Supplemental Figure 9. **Effect of SIV infection and ART on lipid profiles.** Longitudinal changes in serum levels of total cholesterol (A), triglycerides (B), HDL (C), and LDL (D). P values for longitudinal within-group (Time), between-group (Group), and time by group (Time*Group) interaction changes are indicated. Significance determined by mixed-effects analysis with either Tukey's or Sidak's multiple comparison test. *, $p<0.05$; **, $p<0.01$; ***, $p<0.001$. All data are means ± SEM.

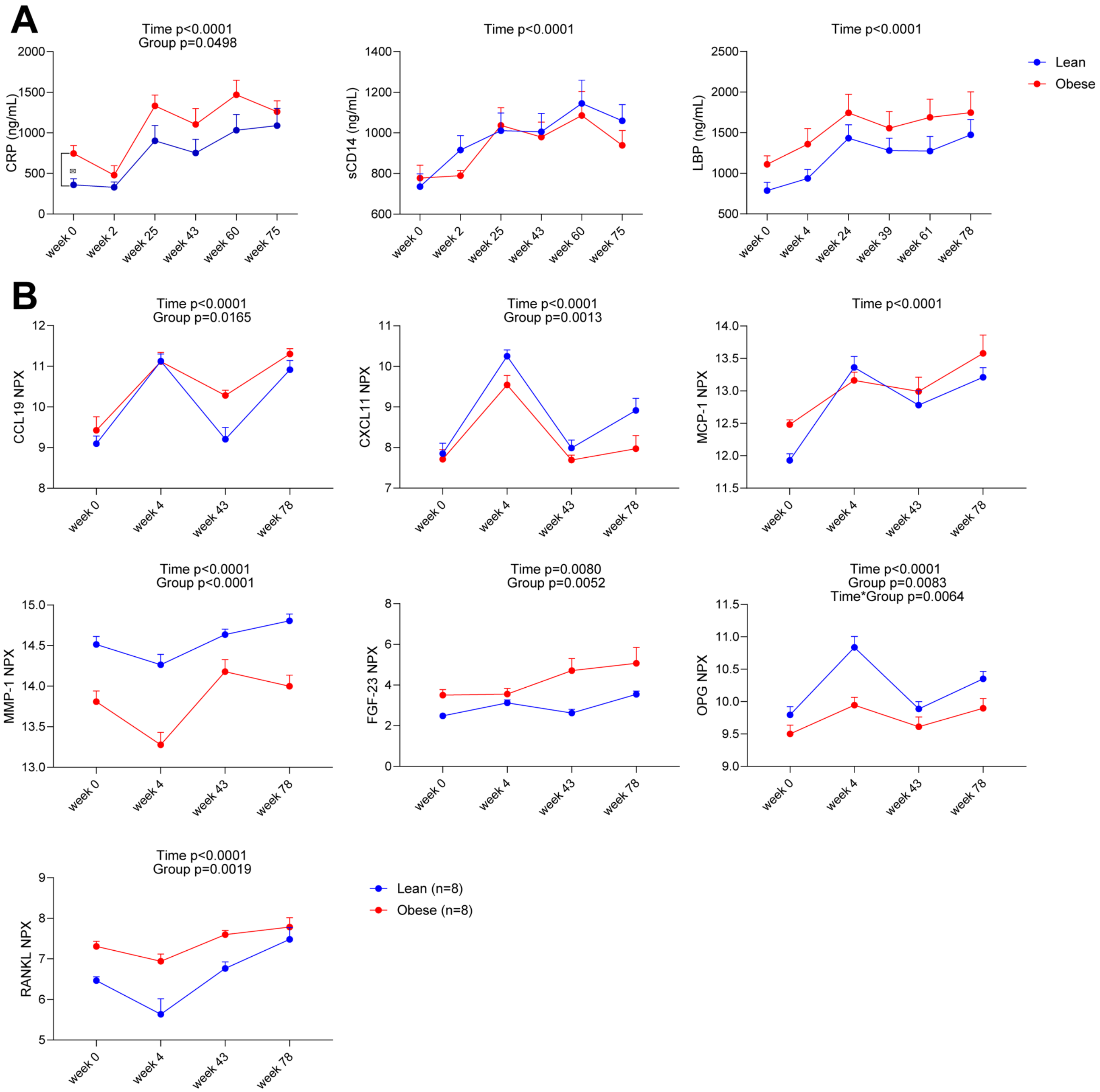

Supplemental Figure 10. **Effect of SIV infection and ART on cytokine profiles**. A. Longitudinal changes in plasma C-reactive protein (CRP), soluble (s)CD14, and serum LPS binding protein (LBP) determined by ELISA. B. Longitudinal changes in plasma CCL19, CXCL11, MCP-1, MMP-1, FGF-23, OPG, and RANKL determined by Olink proximity extension assay. Y axis indicates Log2-scale arbitrary normalized protein expression (NPX) values. Significance determined by two-way ANOVA for p values for longitudinal within-group (Time), between-group (Group) and time by group (Time*Group) interaction changes. All data are means ± SEM.

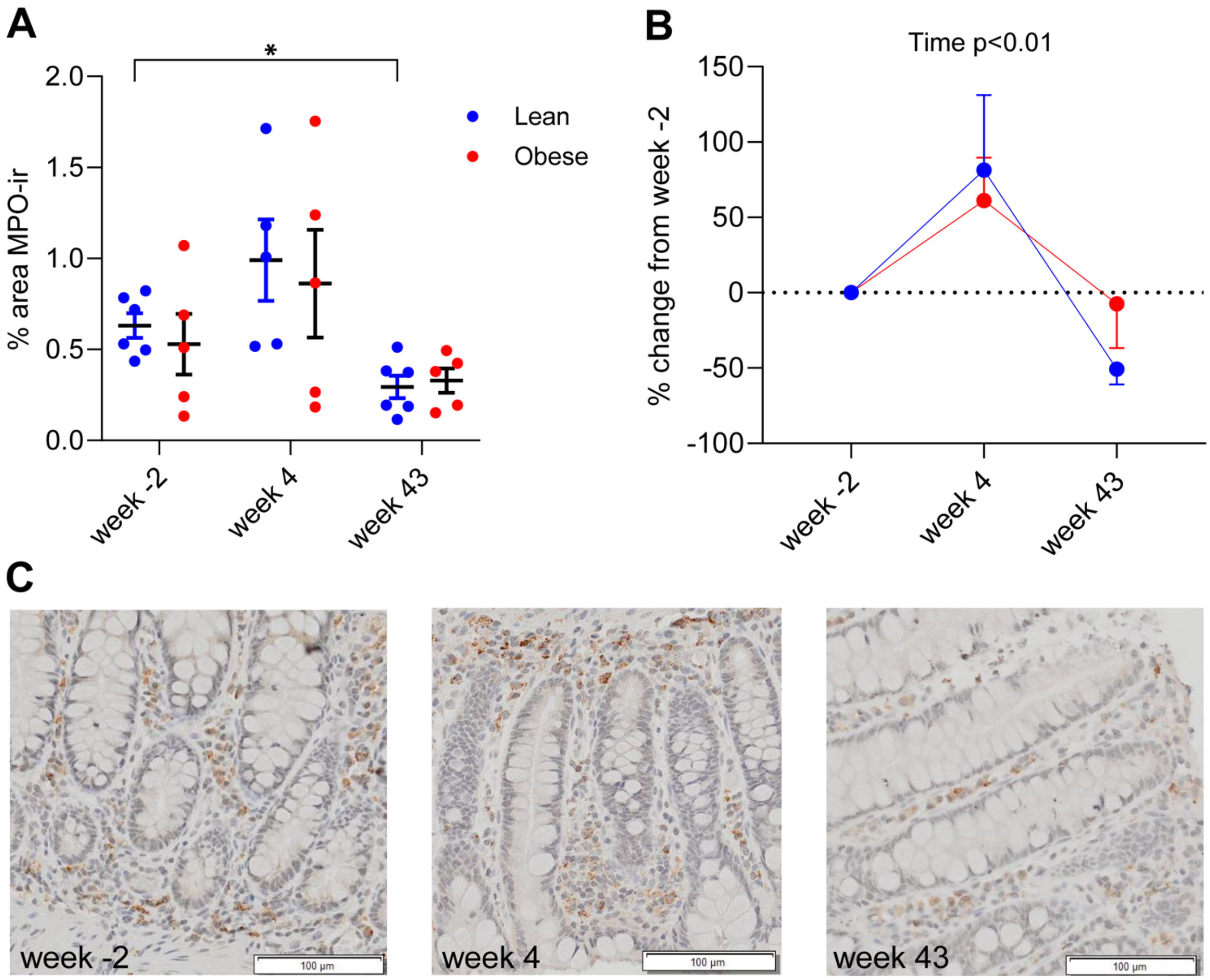

Supplemental Figure 11. **Effect of SIV infection and ART on neutrophil invasion of the colon**. A. Quantitation of myeloperoxidase (MPO)-immunoreactive (MPO-ir) area in ascending colon biopsy samples from lean and obese animals at baseline (week -2), peak viremia (week 4 post-infection) and following ART treatment (week 43 post-infection). B. Longitudinal % change in MPO-ir area. C. Representative images of MPO staining at weeks -2, 4, and 43. *, $p<0.05$. All data are means ± SEM.

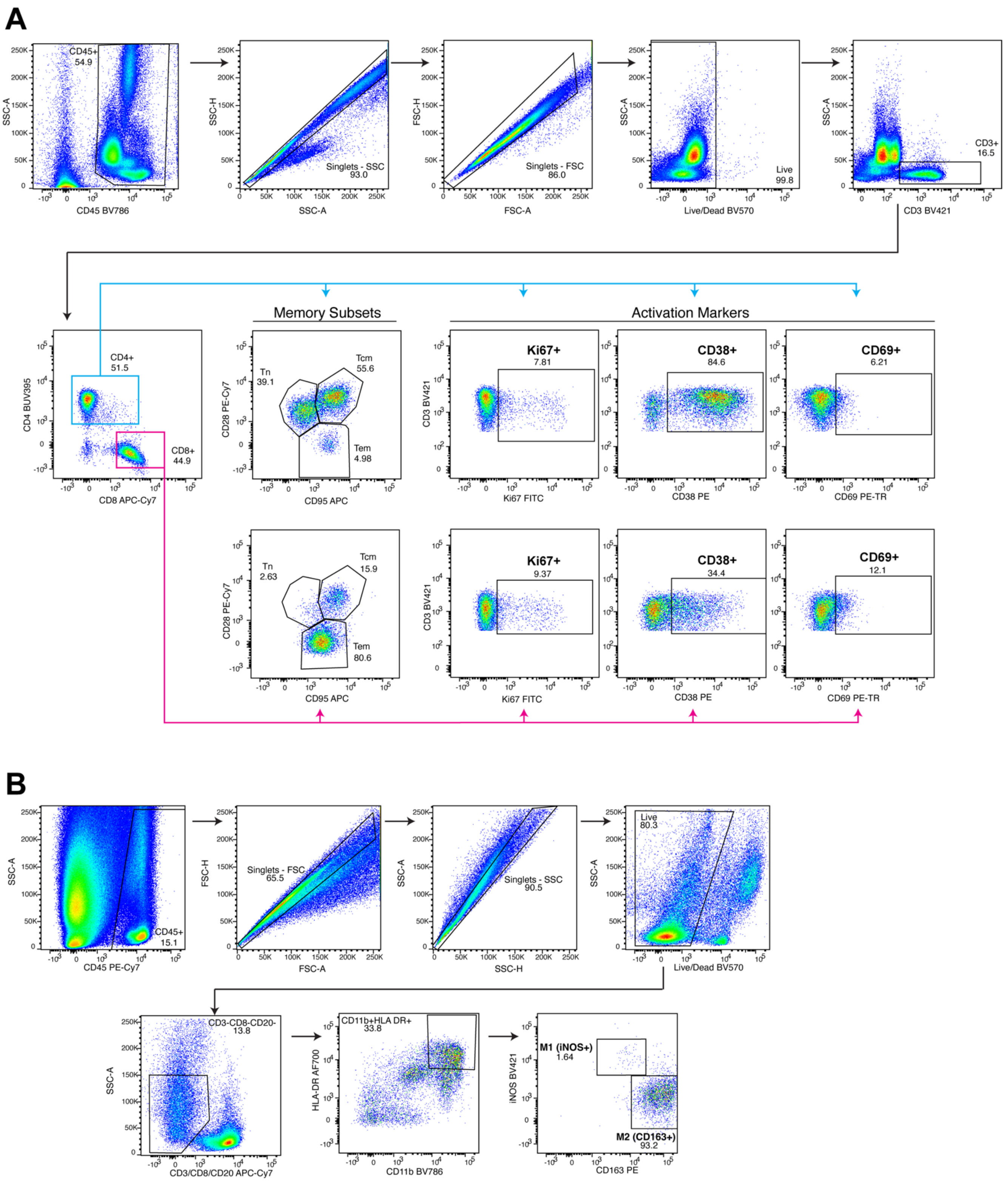

Supplemental Figure 12. **Flow cytometry gating strategies.** A. Gating strategy used to evaluate CD4 +and CD8+ T cell frequency, T cell activation measured by Ki67, CD38, and CD69, and memory subset frequency. B. Gating strategy used to evaluate frequency of M1 (iNos+ CD163-) and M2 (iNos- CD163+) macrophages.

Supplemental Methods

SIV immune responses. IFN-γ ELISpot assays were used to measure the induction kinetics and magnitude of the SIV-specific T-cell response in peripheral blood following SIV infection and ART as previously described (1). Env-binding antibodies were assayed by ELISA using purified SIV Env protein as previously described (2).

Body composition. Determination of total, lean, and fat mass as well as bone mass and as bone mineral content (BMC) was assessed using dual-energy X-ray absorptiometry (DEXA) scanning (Lunar iDXA, GE HealthCare). After an overnight fast, animals were sedated with 3-5 mg/kg Telazol and positioned in a prone position during a standard scan. These scans were typically paired with metabolic tests to decrease number of sedations.

iv glucose tolerance test (ivGTT). Glucose tolerance was assessed as previously described (3). ivGTTs were performed prior to infection and at 22, 42, and 76 weeks post-infection. After an overnight fast, animals were administered a glucose bolus (50% dextrose solution) at a dose of 0.6 g/kg via the saphenous vein. Baseline blood samples were obtained prior to the infusion, and serial samples were taken at 1, 3, 5, 10, 20, 40, and 60 min post-infusion via the contralateral saphenous vein. Blood glucose was measured immediately using a hand-held glucometer (FreeStyle Lite Blood Glucose Monitor; Abbot Diabetes Care, Inc., Alameda, CA). Plasma insulin was assayed by the ONPRC Endocrine Technologies Core using a Cobas 411 (Roche Diagnostics) or by ELISA (insulin ELISA, cat# 80-ONSHU-E01.1, ALPCO). C-peptide levels were also assayed by the ONPRC Endocrine Technologies Core using a Cobas 411. Glucose AUC, insulin IAUC, and C-peptide AUC (CAUC) were calculated using T=0 as baseline using GraphPad Prism Software.

iv insulin tolerance test (ivITT). Insulin sensitivity was assessed by ivITT performed within 2 weeks of ivGTTs. Animals were fasted overnight and sedated the next morning with 3-5 mg/kg telazol. Once the animal was fully sedated, a baseline blood sample was collected and measured for glucose as above. Animals were then administered a 1U/ml bolus of diluted insulin (Humulin-R, NDC 0002-8215-01, Lilly USA) at a dose of 0.1 U/kg via the saphenous vein. Sequential blood samples were taken at 1, 3, 5, 7, 9, 11, 13, 15, 20, and 30 min post-infusion via the contralateral saphenous vein and blood glucose measured. The Insulin Sensitivity Index (ISI) was calculated as the change in glucose level from baseline to 15 min (estimated using linear regression from baseline to 15 min) divided by the initial glucose level as previously described (4).

Cytokine profiling. ELISA analyses were performed on longitudinal plasma (CRP, sCD14, adiponectin) or serum (LBP) samples using the following kits according to the manufacturer's instructions: CRP, ALPCO cat# 30-9710S; sCD14, R&D Systems cat# DC140; LBP, Invitrogen cat# EH297RBI; and total adiponectin, ALPCO total adiponectin cat# 80-ADPHU-E01). Serum leptin was assayed by the ONPRC Endocrine Technologies Core by RIA (Millipore cat# HL-81K). Olink profiling of longitudinal plasma samples was performed using the Olink Target 96 inflammation panel at the High Containment Research Performance Core at the Tulane National Primate Research Center.

islet isolation/perifusion. An 18-gauge plastic iv catheter was inserted into the pancreatic duct and the pancreas was inflated with 50-80 mL of RT Media (RPMI 1640 medium (Thermo Fisher cat# 11875-085), 10% FBS, 100U pen/strep) containing 0.6 mg/ml collagenase P (Sigma-Aldrich cat# 11213865001). After inflation, fat and connective tissue was removed, and the tissue was divided into 12 equal portions and each portion was placed in a 50-ml conical tube containing 11 ml of RT medium containing 0.06 mg/ml collagenase P and incubated in a 37°C water bath for 27 min with agitation every 10 min. After digestion, collagenase was poured off, and 10 ml of RT media was added and shaken for 1 min to disrupt remaining connective tissue. Undigested material was removed by straining through a 500-μm filter and islets were washed 3 times with 50 ml of RT media, followed by a 10-min incubation with bovine DNase I (Sigma-Aldrich, 0.8 U/ml) incubation at 37°C. Islets were

cultured overnight at 37°C/5%CO_2 in RT media with bovine DNase I (Sigma-Aldrich, 0.4 U/ml). After overnight recovery, islets were transferred into prepared columns and placed in a perifusion system (Biorep Technologies Model PERI-4.2) maintained at 37°C. Islets were pre-incubated in Krebs-Ringer bicarbonate HEPES buffer (KRBH) containing 4.0 mM glucose for one hour at a flow rate of 100 μl/min. After pre-incubation, islets underwent alternating 15-minute washes in 4.0 or 16.7 mM glucose-supplemented KRBH for a total of 45 min, with collections of flow-through every three minutes. All perifusion assays were done in triplicate. Collections were stored at 4°C until being assayed for Insulin by ELISA as described above.

Pancreas immunohistochemistry. 5-μm paraffin-embedded sections from the pancreatic tail were deparaffinized and underwent antigen retrieval using HIER buffer L (Epredia cat# TA-999-DHBL). Following permeabilization and blocking in 0.4% Triton-X solution containing 2% normal donkey serum, sections were stained with the following primary antibodies (also shown in **Table S1)**: mouse anti-insulin (Santa Cruz cat# sc-377071, 1:500) or guinea pig anti-glucagon (Millipore cat# 4031-01F, 1:5000) for 24 hr at room temperature. Immunoreactivity was detected by incubation with Alexa secondary antibodies against mouse, guinea pig, or sheep IgG, respectively (Thermo Fisher, 1:1000). 20x fluorescent images were obtained using an Olympus VS110 slide scanner and analyzed with HALO software (Indica Labs). Insulin and glucagon immunoreactivity was quantified using the Area Quantification FL v2.3.4 module.

Lipid levels. Serum levels of total, HDL, and LDL cholesterol and triglycerides were determined using a Horiba ABX Pentra 400 Clinical Chemistry System (Horiba Medical).

Colon immunohistochemistry. 5-μm paraffin-embedded sections of ascending colon biopsies (collected at weeks -2, 4, and 43) were deparaffinized and underwent antigen retrieval using HIER buffer L (Epredia cat# TA-999-DHBL). Sections were stained for myeloperoxidase using a rabbit anti-myeloperoxidase (MPO) antibody (Agilent cat# A039829-2, 1:2000; also shown in **Table S1**) in an Autostainer 360 (Thermo Fisher), and staining was detected with the DAB Ultravision Quanto detection system (Epredia cat# TL-060-HD). 20x brightfield images were obtained using an Olympus VS110 slide scanner, and the density of MPO staining was quantified with HALO software using the Area Quantification module v2.4.2.

Supplemental References

1. Anderson B, et al. FGF21 decreases body weight without reducing food intake or bone mineral density in high-fat fed obese rhesus macaque monkeys. Int J Obes. 2018;42:1151-1160.

2. Comstock SM, et al. High-fat diet consumption during pregnancy and the early post-natal period leads to decreased alpha cell plasticity in the nonhuman primate. Molecular Metabolism. 2012;2:10-22.

3. Webb GM, et al. The human IL-15 superagonist ALT-803 directs SIV-specific CD8(+) T cells into B-cell follicles. Blood Advances. 2018;2:76-84.

4. Shapiro MB, et al. Single-dose bNAb cocktail or abbreviated ART post-exposure regimens achieve tight SHIV control without adaptive immunity. Nat Commun. 2020;11:70.
